# Development of light-responsive protein binding in the monobody non-immunoglobulin scaffold

**DOI:** 10.1038/s41467-020-17837-7

**Published:** 2020-08-13

**Authors:** César Carrasco-López, Evan M. Zhao, Agnieszka A. Gil, Nathan Alam, Jared E. Toettcher, José L. Avalos

**Affiliations:** 1 Department of Chemical and Biological Engineering, Princeton University, Princeton, NJ 08544 USA; 2 Department of Molecular Biology, Princeton University, Princeton, NJ 08544 USA; 3 Andlinger Center for Energy and the Environment, Princeton University, Princeton, NJ 08544 USA

**Keywords:** Proteins, Optogenetics, Protein design

## Abstract

Monobodies are synthetic non-immunoglobulin customizable protein binders invaluable to basic and applied research, and of considerable potential as future therapeutics and diagnostic tools. The ability to reversibly control their binding activity to their targets on demand would significantly expand their applications in biotechnology, medicine, and research. Here we present, as proof-of-principle, the development of a light-controlled monobody (OptoMB) that works in vitro and in cells and whose affinity for its SH2-domain target exhibits a 330-fold shift in binding affinity upon illumination. We demonstrate that our αSH2-OptoMB can be used to purify SH2-tagged proteins directly from crude *E. coli* extract, achieving 99.8% purity and over 40% yield in a single purification step. By virtue of their ability to be designed to bind any protein of interest, OptoMBs have the potential to find new powerful applications as light-switchable binders of untagged proteins with the temporal and spatial precision afforded by light.

## Abstract (web summary)

The ability to reversibly control monobody binding affinity would find use in biotechnology and research applications. Here the authors fuse the light-sensitive AsLOV2 domain to a monobody against the Abl SH2 domain to obtain a light dependent monobody and apply it in vitro and in mammalian cells.

## Introduction

Monobodies^1^ and other synthetic non-immunoglobulin protein-binding scaffolds, such as affibodies, anticalins, and DARPins, bind to their targets with affinities and selectivities typically found in antibodies^2–5^, yet they are much simpler in structure. Because of their ability to be designed to bind a variety of proteins of interest, monobodies have become invaluable tools for biomedical research and biotechnology^6–9^. Monobodies also show great promise as diagnostic tools and future therapeutics, including for autoimmune diseases^10^, cancer^11^ and most recently SARS-CoV-2^12^. The impact of monobodies stems from their very tight binding to highly specific targets. However, their repertoire of applications could be greatly expanded if they were engineered with optogenetic control over their binding affinities, such that they could bind their targets instantly and reversibly depending on light conditions, while maintaining their characteristically high affinity and selectivity.

Monobodies are synthetic proteins derived from the 10th domain of human fibronectin type III. While they were originally designed to functionally resemble nanobodies^1,6,13^, monobodies feature unique structural advantages such as a reduced size (20–25% smaller) and a compact protein core without disulfide bridges^1,7^. Their small size (<100 amino acids), simple structure, and relative stability allows them to be expressed in many cell types and be active inside^14^ and outside of cells^15^. Furthermore, because of their human origins, the use of monobodies as biologic drugs is expected to have a lower risk of unwanted immune responses^16,17^. As synthetic proteins, they offer flexibility in the design and configuration of their binding surfaces, thereby offering alternative paratope-like regions. This has allowed the development of several monobody libraries that vary particular combinations of loops and strands to produce monobodies with different binding modes, including the original *loop-binding*^1,8^ and alternative *side-binding* modes^7,8^. Because of all these advantages we identified monobodies as preeminent synthetic protein binders and ideal candidates in which to engineer light-switchable binding control.

**Fig. 1:**
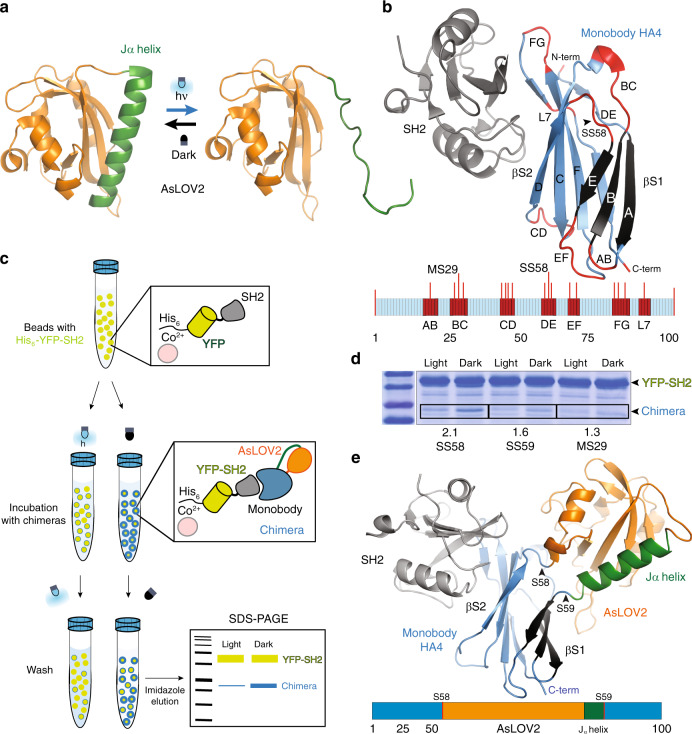
Design and screens of AsLOV2-monobody chimeras and final OptoMB. **a** Light-triggered conformational change of the Jα helix (green) of AsLOV2. **b** Crystal structure of monobody HA4 (blue) bound to SH2 domain (gray) in an archetypal side-binding mode. The monobody fold consists of two β-sheets, βS1(black) and βS2 (light blue), and structurally conserved loops (red, L7 in some monobodies is part of Loop FG), where AsLOV2 was inserted in our chimeras. Loop DE, where AsLOV2 is inserted in OptoMB, is shown with an arrow. The cartoon below shows the relative size and location of loops (red), including positions of AsLOV2 insertion (red lines) and insertion sites of identified light-responsive chimeras: MS29 and SS58 (in OptoMB). **c** Schematic diagram of pull-down screens used to identify light-responsive chimeras. Co^2+^ agarose beads (pink) were used to immobilize His_6_-YFP-SH2 (yellow-gray), which were then incubated with HA4-AsLOV2 chimeras (blue–orange) in either dark or light. After washing and eluting with imidazole, the eluents were resolved on SDS-PAGE to screen for differences in protein band intensity between samples exposed to different light conditions, which reflect differences in chimera SH2 binding. **d** Chimera pull-down screen results showing SDS-PAGE protein bands of two chimeras that bind better in the dark than in the light. These light-responsive chimeras have AsLOV2 inserted in either Loop BC (MS29) or Loop DE (SS58, SS59); the fold difference in band intensity between dark and light binding conditions is shown for each chimera (the complete SDS-PAGE gel of this pull down is shown in Supplementary Fig. 1a). Pull-downs were repeated at least twice observing similar results. **e** Energy-minimized structural model of the dark conformation of OptoMB with AsLOV2 (orange with the Jα helix in green) inserted in position SS58 of HA4 (blue and black), interacting with the SH2 domain (gray). Source data are provided as a Source Data file.

A protein domain widely used for optogenetic tool development is the second light, oxygen, and voltage (LOV) domain from the oat, *Avena sativa*, photosensor Phototropin 1, called AsLOV2^18^. This domain elicits its light response through a large conformational change (Fig. 1a) of its C-terminal Jα helix. In the dark, the Jα helix is packed against the core of the protein^19,20^. However, exposure to blue light (optimally 447 nm) induces the formation of a covalent bond between a photoexcited flavin mononucleotide (FMN) chromophore and a conserved cysteine, which causes the Jα helix to undock, become disordered, and move away from the core domain^19,20^. Back in the dark, the FMN-Cys covalent bond decays, allowing the Jα helix to fold back into its tightly packed dark-state conformation^21,22^. Several studies have exploited this light-triggered conformational change to confer light dependence on natural protein functions. Insertion of AsLOV2 into solvent-exposed loops of kinases, phosphatases, guanine exchange factors, and the small CRISPR inhibitor AcrIIA4 results in light-triggered allosteric switches that make these protein activities and their downstream events light controllable^23,24^. This versatility of AsLOV2 to bestow light-dependent functionalities in a variety of protein contexts encouraged us to use it to engineer light controls in a monobody.

Here we show that by fusing a monobody to AsLOV2 we obtain a light-dependent monobody, or OptoMonobody (OptoMB), whose binding affinity to its cognate protein target is controllable with light. Taking a structure-based protein engineering approach, we inserted AsLOV2 into structurally conserved, solvent exposed, loops of a monobody that binds the SH2 domain of Abl kinase^25^. We show that one of these chimeras preferentially binds to the SH2 domain in the dark, allowing us to reversibly control its binding both in vitro and in mammalian cells. We then harness the ~330-fold change in binding affinity between lit and dark states to implement light-based protein purification^26^ using an OptoMB resin in what we call “light-controlled affinity chromatography” (LCAC). This work represents the first demonstration of light control over the binding activity of a monobody and the first example of this capability in a synthetic non-immunoglobulin protein binder. This class of light-responsive protein binder, which in principle can be engineered, screened, or selected to bind any protein of interest, has great potential for numerous new applications in biotechnology, synthetic biology, and basic research.

## Results

### OptoMonobody design and selection

To demonstrate the feasibility of developing a light-sensitive monobody, we chose the HA4 monobody, reported to bind with high affinity (*K*_d_ ~ 7 nM) to the SH2 domain of the human Abl kinase, in vitro and in cells^25^. This is an interesting and valuable target, as many proteins containing SH2 domains in general, and Abl kinase in particular, are involved in human health and disease^27–29^. In addition, the availability of the crystal structure of HA4 bound to SH2 domain^25^ is a valuable resource for our rational protein engineering approach. Our strategy to develop a light-sensitive HA4 was to design various chimeras of this monobody with AsLOV2 from *A. sativa*, and test their ability to bind and release the SH2 domain depending on light conditions.

To build our chimeras, we inserted a shortened AsLOV2 domain^30^ in all seven structurally conserved, solvent-exposed loops of HA4 (Fig. 1b). Given the large conformational change of AsLOV2 triggered by light (Fig. 1a), our hypothesis was that the native conformation of the monobody domain in some chimeras would be preserved in the dark, allowing it to bind to SH2, but disrupted in the light, causing it to release its target. Guided by the crystal structure of HA4 bound to SH2 (PDB ID: 3k2m)^25^, and considering the side-binding mode of the HA4-SH2 interaction (Fig. 1b), we explored potential sites within the seven solvent-exposed loops in HA4 where we could insert AsLOV2. We selected as many positions as possible in each loop, avoiding those where we have reasons to believe the dark-state conformation of AsLOV2 would disrupt the core β-sheets of the monobody or interfere with its side-binding mode of interactions with SH2. We also excluded positions where the light-triggered conformational change of the AsLOV2 Jα helix might be impeded by clashes with the monobody core. After this structural analysis, we selected 17 AsLOV2 insertion sites across all solvent-exposed loops of HA4, as well as N- or C-terminal fusions (Fig. 1b, Supplementary Table 1).

To find chimeras that can bind the SH2 domain in the dark but not in the light, we screened our constructs using an in vitro pull-down assay (Fig. 1c). First, we produced an N-terminally His-tagged fusion of yellow fluorescent protein (YFP) and SH2 domain (His_6_-YFP-SH2) in *Escherichia coli*, and immobilized it onto cobalt-charged agarose beads. We then incubated the beads with crude extracts of *E. coli* expressing each of the different AsLOV2-HA4 chimeras, in either blue light or darkness. After washing the beads under the same light conditions (see Methods), we eluted with imidazole and resolved the products with denaturing polyacrylamide gel electrophoresis (SDS-PAGE) to analyze the binding of each chimera in different light conditions (Fig. 1c, d, Supplementary Fig. 1). We anticipated that chimeras that bind to SH2 preferentially in the dark would show a more intense band on SDS-PAGE for samples that were incubated and washed in the dark, relative to the samples treated in the light (Fig. 1c).

We found that AsLOV2 insertions in two different HA4 loops produce chimeras with the expected behavior in our pull-down assays (Fig. 1d). One promising chimera has AsLOV2 inserted between residues Met29 and Ser30 (a site we call MS29, following a naming system for sites used in this study), located in Loop BC of HA4 (Fig. 1b, d, Supplementary Fig. 1). We only saw an effect involving this loop when AsLOV2 was inserted at position MS29 and Loop BC was shortened by removing the three surrounding amino acids (Ser30, Ser31, and Ser32, see Supplementary Note 1). Another chimera with positive results has AsLOV2 inserted between Ser58 and Ser59 (site SS58) located in Loop DE of HA4 (Fig. 1b, d). Insertions at other positions within Loop DE (^57^YSSS^60^) show smaller degrees of variation in band intensity between beads treated in the light versus in the dark (Fig. 1d, Supplementary Fig. 1). This suggests that Loop DE is a “hot spot” for favorable orientations between AsLOV2 and the monobody to produce light-responsive chimeras that switch between a conformation that allows target binding (in the dark) and one that promotes target dissociation (in the light). Compared to AsLOV2 insertions at other positions in Loop DE, the insertion at SS58 shows the largest difference between light and dark conditions, with a band that is approximately 2.1 times more intense in dark compared to light (Fig. 1d, Supplementary Fig. 1). Therefore, we chose this chimera to continue our study, naming it αSH2 OptoMonobody (OptoMB). A structural model of this OptoMB (Fig. 1e, Supplementary Movie 1) reveals that the orientation of AsLOV2 in this chimera (modeled in the dark state) is compatible with SH2 binding and suggests a possible mechanism by which a light-triggered conformational change of the Jα helix may disrupt the HA4 monobody to disfavor SH2 binding (see “Discussion”).

### In vitro characterization of OptoMB

**Fig. 2:**
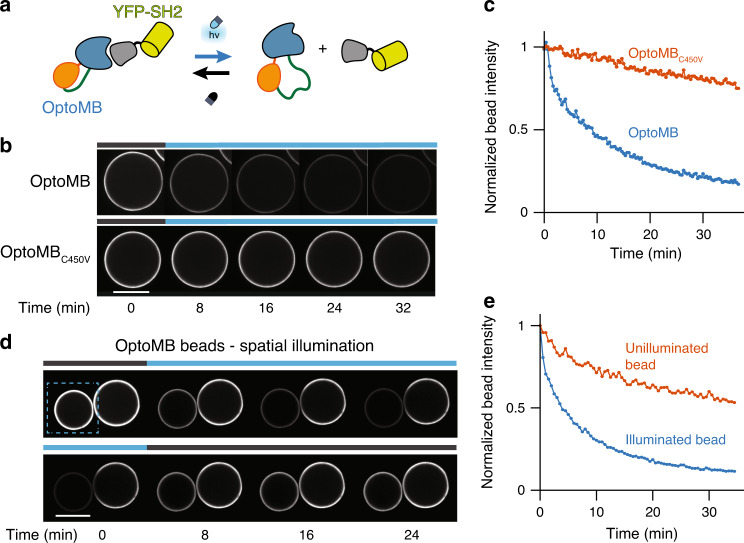
In vitro characterization of OptoMB using agarose beads. **a** Schematic diagram of OptoMB and YFP-SH2 darkness-dependent interaction. In the dark, OptoMB binds to the SH2 domain of YFP-SH2 fusion; upon blue light stimulation, the AsLOV2 domain disrupts the HA4 domain of OptoMB, reducing its affinity for SH2. **b** Time course of fluorescence microscopy images of YFP-SH2 binding to agarose beads conjugated with OptoMB (top panel) or OptoMB_C450V_ as control (lower panel). Starting with beads incubated in the dark, time course begins upon blue light stimulation (*t* = 0), followed by a sequence of images taken every 8 min for a total of 32 min (see Supplementary Movie 2). The bar on top of each time course indicates when the illumination (450 nm) is on (blue) or off (gray) **c** Quantification of the time course fluorescence microscopy experiments shown in **b** for the beads coated with OptoMB (blue) and the ones coated with the light-insensitive control OptoMB_C450V_ (orange). **d** Light-enabled spatial control of OptoMB binding demonstrated in two OptoMB-coated agarose beads incubated in a circulating solution of YFP-SH2. The blue rectangle in the left panel indicates the area used to illuminate only one bead. The top panel shows YFP-SH2 unbinding in illuminated area as beads go from darkness to 24 min of illumination. The bottom panel shows illuminated bead recovery (reversibility) after turning off the blue light for another 24 min (see Supplementary Movie 3). **e** Quantification of spatially controlled binding experiments of YFP-SH2 to two OptoMB-coated agarose beads, shown in **d**, showing normalized YFP fluorescence intensity of both illumined (blue) and unilluminated (orange) beads over time. Scale bars (white) represent 100 μm.

We next set out to test whether the light-dependent interaction of our OptoMB and SH2 domain (Fig. 2a) could be directly visualized by fluorescence microscopy. We cloned and purified His-tagged OptoMB and a variant harboring a mutation in AsLOV2 (V416L) that extends the lifetime of the photoactivated state from 55 to 4300 s^31^ (OptoMB_V416L_) and immobilized them separately onto Ni-charged agarose beads. We also prepared a dark-state OptoMB mutant with the well-characterized C450V mutation in AsLOV2 that prevents light-induced conformational switching (OptoMB_C450V_)^32^. Immobilized OptoMB_C450V_ and parental HA4 monobodies were used as controls. The monobody-coated beads were then incubated with a fusion of YFP and SH2 (YFP-SH2) in the dark until they reached equilibrium and imaged over time using confocal microscopy as we changed conditions from darkness to blue light (450 nm). For both OptoMB and OptoMB_V416L_-coated beads, light exposure induces a pronounced decrease in YFP signal on the surface of the bead (Fig. 2b, c; Supplementary Fig. 2a, Supplementary Movie 2), whereas identical illumination and imaging conditions produced only slow photobleaching for beads coated with OptoMB_C450V_ (Fig. 2b, c, Supplementary Movie 2) or parental HA4 monobodies (Supplementary Fig. 2a). Localized illumination could also be used to restrict SH2 unbinding to a single OptoMB-coated bead in a crowded field. In this case, YFP fluorescence was rapidly and reversibly controlled for the illuminated bead but not a nearby unilluminated bead (Fig. 2d, e, Supplementary Movie 3). These results demonstrate that OptoMBs provide spatiotemporal control over protein binding.

**Fig. 3:**
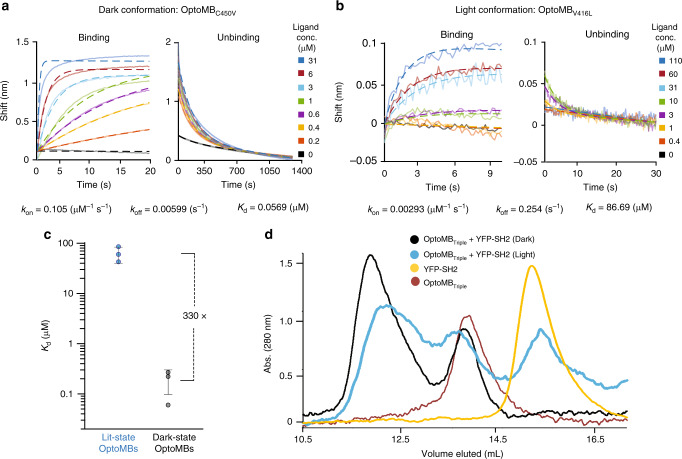
OptoMB binding kinetics and SEC profile for lit and dark states. **a**, **b** Representative BLI measurements of binding (left) and unbinding (right) of YFP-SH2 to immobilized OptoMB. **a** Dark-state measurement using the light-insensitive OptoMB_C450V_, and no external light source. **b** Lit-state measurement using the lit-stabilized OptoMB_V416L_ and exposing the sample to light. The calculated rate (*k*_on_, *k*_off_) and dissociation (*K*_d_) constants are shown below the BLI data. **c** Change in calculated *K*_d_ values between all dark-state (OptoMB_C450V_) and lit-state (OptoMB_V416L_) measurements. Error bars indicate mean ± SD for *n* = 3 independent measurements (see Table 1). **d** Size exclusion chromatography profiles of SUMO-tagged OptoMB_V416I_G528A_N538E_ (OptoMB_Triple_) and YFP-SH2 interactions in light (blue line) and dark (black line), compared to pure OptoMB_Triple_ (red) or YFP-SH2 (yellow) controls. Source data are provided as a Source Data file.

To quantify the changes in OptoMB-SH2 binding, we determined the kinetic rate constants and binding affinity in different light conditions. In these assays we took advantage of the different classes of mutations in AsLOV2 mentioned above to vary properties of the OptoMB binding switch. Bio-layer interferometry (BLI) uses visible light for measuring changes in binding, so we used the light-insensitive OptoMB_C450V_ variant for assessing dark-state-binding kinetics and affinity. Conversely, we ensured that illumination could drive efficient conversion to the lit state by using the OptoMB_V416L_ variant. We fit the resulting binding and dissociation data to a mass-action kinetic-binding model (Fig. 3a, b, Supplementary Fig. 2b), from which we obtained estimates of the rate constants of binding (*k*_on_) and unbinding (*k*_off_) as well as the overall dissociation constant (*K*_d_) of the OptoMB-SH2 interaction in different light conditions (Table 1; Supplementary Table 2, Supplementary Fig. 2c, d).

**Table 1:** Rate and dissociation constants from BLI experiments.

Variant	State measured	*k*_on_ (μM^−1^ s^−1^)	*k*_off_ (s^−1^)	*K*_d_ (μM)
Monobody HA4	–	0.0631	0.0145	0.23
OptoMB	Light conformation	<0.001	0.21 ± 0.09	>100
^a^OptoMB_C450V_	Dark conformation	0.071 ± 0.033	0.01 ± 0.004	0.19 ± 0.11
^a^OptoMB_V416L_	Light conformation	0.004 ± 0.001	0.23 ± 0.02	63 ± 23

^a^Average of three individual measurements ± SD.

We found that the binding affinity of OptoMB to SH2 changes dramatically when switching from dark to light conditions. The average dissociation constant of the OptoMB-SH2 interaction in the dark (OptoMB_C450V_) is *K*_d_ = 0.19 ± 0.11 μM (mean ± SD), which is comparable to our measurements for the HA4 monobody (Table 1, Supplementary Fig. 2c, d). However, in the light (OptoMB_V416L_) it is drastically increased to *K*_d_ = 63 ± 23 μM (Table 1, Supplementary Fig. 2c). This amounts to an ~330-fold-change in binding affinity between light conditions (Fig. 3c), which explains the light-dependent behaviors observed in the bead-imaging experiments above. The change in *K*_d_ of the lit state arises equally from a decrease in the binding rate constant (*k*_on_) and an increase in the unbinding rate constant (*k*_off_) (Table 1, Supplementary Table 2, Supplementary Fig. 2c, d). These data are consistent with a light-induced change in the conformation of the OptoMB that disrupts the binding interface, substantially decreasing the likelihood of a productive association between the lit-state OptoMB and its target SH2, and equally increasing the likelihood of dissociation of the bound complex.

The light dependency of OptoMB interactions with YFP-SH2 can also be analyzed in solution using size exclusion chromatography (SEC). For this demonstration, we used an additional OptoMB variant with a V416I mutation in AsLOV2, which extends the lifetime of the lit state up to 821 s^33^, as well as two other mutations, G528A and N538E, reported to stabilize the dark-state conformation^34^. This triple mutant (OptoMB_V416I_G528A_N538E_ or OptoMB_Triple_) has stabilized lit and dark states, lower leaky activation in the absence of illumination, and thus better overall dynamic range of photoswitching^33,34^, which we expected would improve the performance of OptoMB in packed chromatography columns. BLI experiments confirmed that this mutant has a reduced binding affinity in the lit state relative to OptoMB_V416L_ (Supplementary Table 2, Supplementary Fig. 2b, c), and thus at least the same change in *K*_d_ in different light conditions as this variant (Fig. 3c).

We prepared mixtures of purified YFP-SH2 and OptoMB_Triple_ (SUMO tagged to boost expression, see “Methods”), in which the OptoMB_Triple_ (or HA4 monobody, as control) was added in excess (see “Methods”). Each mixture was then run in a gel filtration column under continuous darkness or blue light (Supplementary Fig. 3a), with pure samples of OptoMB_Triple_, YFP-SH2 and HA4, as well as YFP-SH2/HA4 mixtures run under different light conditions as controls (Supplementary Fig. 3b, c). When run with OptoMB_Triple_ in the dark or with the parental HA4 monobody, YFP-SH2 elutes as a monobody-bound complex without exhibiting a peak for unbound YFP-SH2 protein (Fig. 3d, Supplementary Fig. 3c). In contrast, the illuminated OptoMB_Triple_ mixture displays a peak corresponding to the monomeric YFP-SH2 as well as a longer average retention time for the OptoMB-target complex (Fig. 3d). Taken together, the light-triggered reduction in OptoMB-SH2 binding observed in solution and protein-coated surfaces is consistent with the two-order of magnitude change in binding affinity between dark and light conditions measured with BLI, laying the ground for the following in vitro application.

### Light-controlled affinity chromatography

**Fig. 4:**
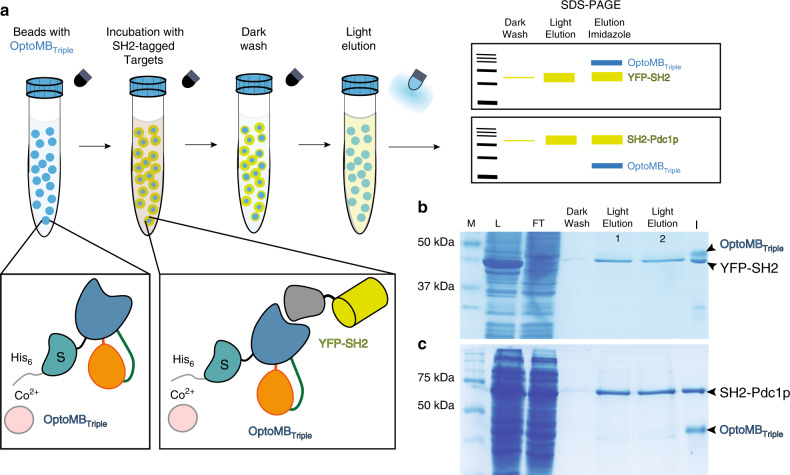
Light-controlled affinity chromatography (LCAC) to purify SH2-tagged proteins using cobalt-immobilized OptoMB. **a** Schematic diagram of functionalization of LCAC Co^2+^-agarose beads using His_6_-OptoMB_V416I_G528A_N538E_ (OptoMB_Triple_) containing a SUMO tag (S) and LCAC procedure involving dark incubation of crude *E. coli* extract containing YFP-SH2, washing in the dark, and eluting with blue light in batch or column (Supplementary Fig. 4). SDS-PAGE was used to resolve the fractions from each purification step. **b**, **c** SDS-PAGE gel of LCAC-purified YFP-SH2 (**b**) and SH2-Pdc1p (**c**). Molecular weight markers (M), lysate (L), unbound flow through (FT), washing step in the dark (Dark Wash), two consecutives light elution aliquots (Light Elution 1 and 2), and the imidazole elution (I) were resolved in 12% SDS-PAGE. Purifications were repeated at least three times observing similar results.

We reasoned that the substantial change in OptoMB binding affinity in light could open the door to purifying a protein of interest simply by shifting illumination conditions (Fig. 4a), a procedure that we termed “light-controlled affinity chromatography” (LCAC). We immobilized two variants of His-tagged OptoMBs harboring either the wild-type AsLOV2 or the triple mutant (SUMO-tagged OptoMB_Triple_) described above, onto Co^2+^-Agarose beads to make αSH2-OptoMB affinity resins. We then incubated these resins with crude lysate from *E. coli* overexpressing YFP-SH2. After washing thoroughly in the dark (see “Methods”), we eluted with blue light either in batch (Fig. 4b) or in a column (Supplementary Fig. 4). Finally, after light elution, beads were eluted with imidazole to recover any remaining protein bound to the beads in order to estimate the capacity and yields of the resin. With these initial LCAC purification trials, we achieved 95–98% purity in a single step, with yields ranging from 18 to 30%, and binding capacities from 112 to 145 nmol (4.5–6 mg) of SH2-tagged YFP per mL of OptoMB resin, depending on the OptoMB variant used (Table 2).

**Table 2:** Purification parameters of LCAC batch experiments.

Purified protein	OptoMB version	Resin type	Purity^a^ (%)	Resin capacity^a^ (mg/mL)	Yield^a^ (% of recovery from the resin)
YFP-SH2	OptoMB	Talon^b^	98.25 (±2.26)	5.85 (±0.67)	17.8 (±2.18)
YFP-SH2	^c^OptoMB_Triple_	Talon^b^	95.32 (±0.46)	4.5 (±0.33)	29.79 (±3.28)
SH2-Pdc1p	^c^OptoMB_Triple_	Talon^b^	95.53 (±1.05)	5.24 (±0.52)	39.01 (±2.61)
YFP-SH2	^c^OptoMB_Triple_	CNBr^d^	99.78 (±0.22)	2.55 (±0.22)	40.29 (±3.02)
SH2-Pdc1p	^c^OptoMB_Triple_	CNBr^d^	96.69 (±1.24)	1.55 (±0.18)	42.28 (±4.34)

Values expressed as mean ± SD for *n* = 3 independent measurements. Source data are provided as a Source Data file.

^a^See “Methods” for calculations.

^b^OptoMB was His-tag bound.

^c^SUMO tagged.

^d^OptoMB was covalently conjugated.

To test whether LCAC could be applied to larger and more complex proteins, we used it to purify the main pyruvate decarboxylase from *Saccharomyces cerevisiae*, Pdc1p. This enzyme catalyzes the decarboxylation of pyruvate to acetaldehyde for ethanol fermentation, and is composed of a homotetramer of 61 kDa monomers^35^, significantly larger than YFP. We fused the SH2 domain to the N-terminus of Pdc1p (SH2-Pdc1p) and performed LCAC to purify it from crude *E. coli* lysate, as described above, using a resin coated with OptoMB_Triple_. This procedure enabled purification of Pdc1p to 96% purity with a 39% yield (Fig. 4c, Table 2). It is noteworthy that this purification works well despite the potential binding avidity of Pdc1p tetramers, which would be predicted to increase the protein’s apparent affinity for the resin in both the light and dark. These results demonstrate that LCAC can be applied to purify relatively large proteins with quaternary structures of up to at least 300 kDa (including the fused SH2 domain), achieving a high degree of purity and an acceptable yield. Our results with YFP-SH2 and SH2-Pdc1p further demonstrate that OptoMB-assisted purification is compatible with both N- and C-terminal SH2 tags.

While metal-affinity beads are effective at immobilizing OptoMB for LCAC (Fig. 4, Supplementary Fig. 4), they may be incompatible with some protein purification methods. Thus, to determine whether an alternative resin could be used for LCAC, we immobilized our OptoMB_Triple_ onto cyanogen bromide-activated sepharose beads (CNBr beads), which immobilizes proteins by making covalent bonds with their primary amines (see “Methods”). Following the same purification protocol as above, we found that CNBr beads are also effective at purifying both YFP and Pdc1p (Supplementary Fig. 5). A single step of CNBr-based purification achieved yields above 40% and purity of 96.7–99.8%, surpassing any other LCAC method tested (Table 2). These gains are likely related to the lower non-specific binding of *E. coli* proteins to CNBr-sepharose relative to Co^2+^-agarose beads, and the covalent attachment of OptoMB, which allows for more efficient and extensive washing at higher salt concentrations. However, the total loading capacity of the CNBr-OptoMB-sepharose beads is not as high as that of Co^2+^-OptoMB-agarose (Table 2), probably because random crosslinking to the CNBr beads inactivates a substantial fraction of the OptoMB by occluding its binding surface to SH2. Although LCAC could be further improved by optimizing the resin (including for reusability), method, or amino acid sequence of the OptoMB, our results demonstrate the feasibility of a practical in vitro application of light-responsive monobodies for protein purification. This approach would make it possible to use buffer conditions that are optimal for protein stability throughout the purification process without needing to elute with a buffer exchange, which may damage the protein of interest (such as the low pH commonly used in antibody-based purification), or require lengthy and expensive subsequent dialysis. It also opens the possibility of using protein-specific OptoMBs to purify proteins that are difficult to fuse to affinity tags.

### Light-dependent OptoMB binding in cells

**Fig. 5:**
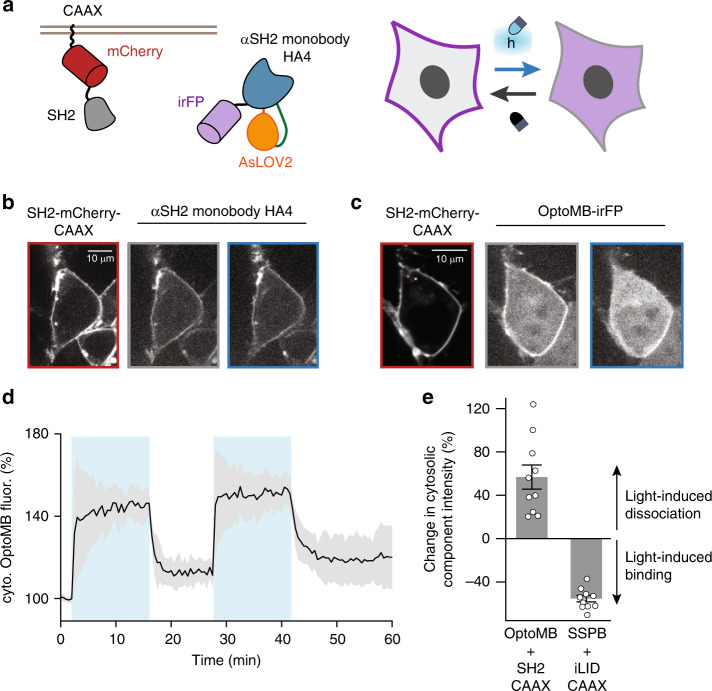
Characterization of OptoMB in mammalian cells. **a** Schematic diagram of the membrane-binding assay used, where irFP-tagged OptoMB (blue–orange–purple) binds to a fusion of SH2 (gray), mCherry (red), and CAAX (black), anchored to the plasma membrane (PM) of HEK293T cells. In the dark, OptoMB-irFP binds to the membrane-bound SH2, enhancing irFP signal at the PM and reducing it in the cytosol. In blue light, OptoMB releases SH2, causing a reduction in irFP signal at the membrane and an increase in the cytosol. **b**, **c** Images represent two replicate experiments. HEK293T cells expressing SH2-mCherry-CAAX and irFP-tagged HA4 monobody (**b**) or OptoMB-iRFP (**c**) imaged in the dark or blue light. Left panels (red) show mCherry fluorescence of SH2 fusion anchored to the PM; central (gray) and right (blue) panels show HA4-irFP (**b**) or OptoMB-iRFP (**c**) fluorescence in the dark and after 30 s of blue light stimulation, respectively, showing irFP-HA4 localized to the PM in either light condition (**b**) or OptoMB enriched at the PM in the dark and in the cytosol in the light (**c**). White scale bar in the cell images is 10 μm. **d** Cytosolic OptoMB-iRFP fluorescence changing over time due to periodic pulses of blue light (blue sections). The fluorescence is expressed in percentage from the original value of cells in the dark. Curve and shaded regions indicate mean ± SD for at least 10 cells respectively. **e** Comparison of light-induced translocation by OptoMB and iLID-SSPB. Error bars indicate mean ± SEM for *n* = 10 cells per variant. For iLID/SSPB translocation, cytosolic BFP fluorescence was measured using NIH3T3 cells with a stably integrated BFP-SSPB-SOScat-P2A-iLID-CAAX lentiviral construct. Source data are provided as a Source Data file.

We have shown that OptoMB-SH2 binding can be controlled with light in vitro; as a final test of this system, we assessed whether similar control can be achieved in live mammalian cells^14^. We transduced HEK293T cells with lentiviral vectors encoding a membrane-localized, fluorescent SH2 target protein (SH2-mCherry-CAAX) and cytosolic fluorescent OptoMB (OptoMB-irFP), reasoning that a light-dependent change in SH2-OptoMB binding would cause the OptoMB to redistribute between the cytosol and plasma membrane (PM) (Fig. 5a), as has been observed for conventional optogenetic protein–protein interactions in previous studies^36–38^. As a control, we expressed irFP-labeled HA4 monobody instead of OptoMB, which would be expected to bind to the membrane-localized SH2-mCherry-CAAX regardless of illumination conditions.

Fluorescence imaging confirmed the PM localization of SH2-mCherry-CAAX (Fig. 5b, c, left panels) as well as constitutively PM-bound HA4-irFP (Fig. 5b). In contrast, we observe a light-dependent shift in OptoMB localization (Fig. 5c), with PM enrichment in the dark and rapid redistribution to the cytosol upon light stimulation. Applying cycles of light and darkness further revealed that light-controlled binding is fully reversible intracellularly (Fig. 5d, Supplementary Movie 4). We also compared the extent of OptoMB cytosol-to-membrane redistribution to the well-established iLID-SSPB optogenetic heterodimerization tool^39,40^. This analysis showed that our OptoMB displays a change in cytosolic intensity between light conditions that is equal in magnitude (both close to 60%) to that of iLID-SSPB, but with opposite sign (Fig. 5e). These experiments demonstrate functional photoswitching of OptoMB binding in cells to a level comparable to existing optogenetic tools, opening the door to their application in this context.

## Discussion

Here we show that by taking a rational protein engineering approach it is possible to develop a light-switchable monobody (OptoMB). By fusing the light-responsive AsLOV2 domain to a structurally conserved loop of the H4A monobody that binds the SH2 domain of human Abl kinase, we developed an OptoMB that shows an ~330-fold drop in binding affinity when changing conditions from darkness to blue light. In comparison, the well-established iLID optogenetic switch displays an ~58-fold change in binding affinity between light conditions^39^. Furthermore, the light responsiveness of OptoMB is reversible and effective at controlling binding to proteins fused to SH2 (at either their N- or C-terminus), both in vitro and in cells. OptoMBs, along with light-switchable nanobodies (OptoNBs)^30^, belong to a class of light-dependent protein binders we call OptoBinders (OptoBNDRs), which offer promising new in vivo and in vitro applications.

A close inspection of the structural model of OptoMB and binding measurements suggest a possible mechanism for the light-dependent binding affinity of OptoMB. The monobody protein fold consists of two antiparallel β-sheets (βS1 and βS2) that interact with each other to form the protein core (Fig. 1b). In our original chimera screens, we inserted AsLOV2 in all intervening loops within (AB, CD, FG, and L7) and between (BC, DE, and EF) these β-sheets, which are accessible in the side-binding mode of monobodies. The only chimeras that show a change in binding affinity in different light conditions are those with AsLOV2 inserted in Loops BC and DE, with DE being the only loop with positive results at multiple insertion sites. Both of these loops connect βS1 and βS2 with each other, suggesting that chimeras involving either loop may act by a similar mechanism, where the light-triggered conformational change of the Jα helix pulls the βS1 and βS2 apart from each other. Chimeras with AsLOV2 inserted in Loop EF located at the opposite side of the βS1–βS2 interaction relative to BC and DE show equally faint SDS-PAGE bands in either light condition (Supplementary Fig. 1b, d), suggesting limited expression in *E. coli*, or weak binding to SH2 independently of light. The consequence of pulling on βS1 and βS2 from Loops BC or DE is probably at least a partial disruption of the interactions and angle between them. This in turn would likely change the curvature of βS2 involved in the side-binding mode of monobodies, which defines the shape of the paratope-like surface of H4A and its specific binding interactions with SH2^25^. Interestingly, because the conserved fold of monobodies always includes βS1 and βS2 interactions^7^, this light-triggered disruption of the binding surface may be transferable to other monobodies with a side-binding mode. It also suggests that mutating residues involved in βS1–βS2 interactions may provide some opportunities to tune the photoswitchable behavior of the OptoMB, by stabilizing either the dark- or lit-state conformations.

This model of light-induced disruption of the monobody’s target-binding site is consistent with our measurements of binding kinetics. We originally hypothesized that light stimulation might strain the OptoMB-SH2 interaction causing them to dissociate without necessarily inducing dramatic changes in binding site accessibility, thus predicting mostly a light-induced increase in the OptoMB-SH2 off-rate (*k*_off_). However, our BLI measurements revealed that both *k*_on_ and *k*_off_ are equally affected in the light (~20-fold change in opposite directions, Table 1, Supplementary Table 2, Supplementary Fig. 2b, c). This suggests a significant disruption of the binding surface of the OptoMB in the light, which not only accelerates the dissociation of the bound chimera but also slows down its binding to the target to begin with. Such disruption would be expected from pulling on the β-sheet interactions that make the core of the monobody fold. However, despite what is likely to be a substantial conformational change in the OptoMB, we find that productive binding is reversible in vitro (Fig. 2d, Supplementary Movie 3) and in mammalian cells (Fig. 5d, Supplementary Movie 4). Overall, these data are consistent with a large change in the overall orientation or conformation of the βS1–βS2 sheets, substantially disrupting binding but without driving irreversible protein misfolding. It is possible that the small size of the monobody domain, the short range of interactions between βS1 and βS2, and the lack of disulfide bonds in the monobody fold facilitate this high efficiency of interconversion between binding states.

Comparison of our binding data to a maximum theoretical value that assumes perfect transmission of energy between AsLOV2 and the monobody’s binding interface suggests that light-induced changes in OptoMB could be further improved. Previous studies have measured the free energy available from the dark-to-light conformational change of AsLOV2 using NMR spectroscopy^41^ and developed analytical models to study equilibrium constants of the lit and dark states of AsLOV2^34^. These studies predict that 3.8 kcal/mol of energy is transmitted from the absorption of one photon to structural rearrangements in AsLOV2. If all of this energy was transmitted to a change in OptoMB binding, it would result in a ~600-fold difference in binding affinity between the lit and dark states. Our BLI experiments measured a change in *K*_d_ values of ~330-fold, which is within the same order of magnitude of this maximum theoretical prediction, indicating that the light-induced conformational change of AsLOV2 is efficiently transmitted to disrupt the SH2 binding surface of the monobody domain. Nevertheless, it also suggests that further improvements might be achieved by optimizing the insertion site or linkers between the AsLOV2 and monobody, or by engineering the H4A domain to improve the light-induced allosteric coupling between the AsLOV2 domain and SH2-binding site. It is also possible that performance for particular applications might be improved by tuning the OptoMB-SH2 affinity to either weaker or tighter values. Finally, we note that the AsLOV2 domain might also be altered to tune the efficiency of the light-induced conformational change, for instance by further stabilizing the lit- or dark-state conformations or by altering its photoswitching kinetics (Table 1, Supplementary Table 2). In principle, these changes could increase the overall energy beyond the 3.8 kcal/mol measured for the wild-type AsLOV2 domain. The structural targets set for optimization and the expected results will of course vary depending on the particular application objectives and the individual OptoMB/ligand pair in question.

Even though the demonstrations used in this study rely on fusing SH2 to different proteins, the true potential of this technology is not so much to replace the light-responsive tags used in previous optogenetic systems with SH2 and OptoMB, but in the possibility of engineering other monobodies against different targets of interest to make their specific interaction light dependent. A few lines of reasoning suggest that this extensibility is likely. While our OptoMB design is based on the side-binding mode present in HA4 (involving the Loop FG, one or more β-strands from βS2 and, occasionally, Loop CD), and is thus unlikely to be applicable to monobodies that present a loop-binding mode (involving Loops BC, DE, and FG), the majority of monobodies that have been structurally characterized (23 out of 32) bind their targets through the side-binding mode (Supplementary Table 3, Supplementary Fig. 6, see Supplementary Note 2). Further evidence suggests that the side-binding mode is more prevalent, as specimens with this binding mode are often identified from libraries in which only the Loops (BC, DE, and FG) were varied (Supplementary Table 3). As a consequence, the side-binding mode has been exploited to generate the so-called *side and loop* libraries that specifically vary the Loop FG, βC, and βD (and Loop CD in some versions), which have yielded monobodies against many targets^7–9,42^ (Supplementary Table 3). Thus, the high frequency of the side-binding mode, which leaves Loops BC and DE unconstrained, and the availability of side and loop libraries that leave these loops constant provide ample opportunities to extend AsLOV2 insertion to additional targets. We can also envision an alternative inverse strategy for obtaining OptoMBs against new targets, using our current OptoMB as a template to generate new side and loop libraries and directly select for photoswitchable binders to distinct targets of interest. Our approach may thus be useful to design reversible interactions to a protein of interest without the need for a binding tag, which for some proteins may be impractical, not possible, or interfere with their natural activity.

## Methods

### Plasmid construction of chimeras for bacterial expression

One-step isothermal assembly reactions (Gibson assembly) were performed using previously described methods^43^. The monobody HA4 and the SH2 domain (codon optimized for *E. coli* expression) were ordered as gBlocks from Integrated DNA Technologies (IDT) containing homology arms. The following vectors from the pCri system^44^ were used: pCri-7b for constructs without a 6x-histidine tag; pCri-8b for constructs with a 6x-histidine tag (N-terminus), and pCri-11b for constructs with both SUMO and 6x-histidine tags (N-terminus). As the pCri vectors contain YFP, the synthetized SH2 domain was inserted into pCri-7b and 8b (previously linearized with *Xho*I) by Gibson assembly to construct EZ-L664 and EZ-L703 (see Supplementary Table 4). Monobody HA4 was inserted into pCri-7b; the vector was digested (opened) with *Nhe*I and *Xho*I and Gibson-assembled to build the template (EZ-L663) used for the AsLOV2 insertions. A stop codon was added before the in-frame (C terminus) 6x-histidine tag of pCri-7b. The AsLOV2 domain (residues 408–543) was either amplified by PCR from previous constructs^30^ (wt AsLOV2) or synthesized as IDT gBlocks (AsLOV2 mutants). To insert AsLOV2 into the monobody, the backbone from the initial construct containing HA4 (EZ-L663) was PCR amplified, using Takara Hifi PCR premix, starting from the insertion positions (Supplementary Table 1) that were selected (and adding homology arms to AsLOV2). Next, chimeras were finally assembled mixing each of the amplified products of the backbone PCR from EZ-L663 with the AsLOV2 domain obtained from either PCR amplification (wt AsLOV2) or synthesized by gBlocks (AsLOV2 mutants). SUMO tags were added by inserting (with Gibson assembly) the PCR product of the full-length chimeras (with homology arms) into the pCri-11b plasmid, previously opened with *Nhe*I and *Xho*I. *PDC1* was amplified from *S. cerevisiae* (S288C) genomic DNA using PCR, also with homology arms, and the construct (EZ-L886) was built via Gibson assembly (3 fragments) with digested pCri-7b (*Nhe*I and *Xho*I) and the PCR product of *SH2* amplified from EZ-L664 (with homology arms). All constructs (Supplementary Table 4) were sequenced by Genewiz and all protein sequences are available in Supplementary Note 1. We used chemically competent DH5α to clone all vectors. After verifying the plasmid sequence, vectors were used to transform chemically competent BL21 (DE3) or Rosetta strains for protein expression.

### Construction of the structural model of OptoMB

To build the structural model of the HA4-AsLOV2 chimera (OptoMB) interacting with the SH2 domain (Fig. 1e), a shortened version^30^ (residues 408–543) of the AsLOV2 domain (PDB ID: 2V1A)^45^ was manually inserted to residues S58 and S59 of the monobody HA4, using the program *Coot*^46^. The crystal structure of HA4 in complex with the SH2 domain (PDB ID: 3k2m)^25^ was used as template. After a manual adjustment, an energy minimization of the HA4-AsLOV2 chimera was carried out with the website version of *YASARA*^47^ (http://www.yasara.org/minimizationserver.htm).

### Plasmid construction for mammalian cells

Constructs for mammalian cell experiments were cloned using backbone PCR and inFusion (Clontech). Monobody HA4 or OptoMB variants were PCR amplified and Gibson-assembled from bacterial plasmids (described above) into a pHR vector with a C-terminal irFP fusion (Addgene #111510). The SH2 domain was amplified from EZ-L664 using PCR and Gibson-assembled into a pHR vector containing a C-terminal mCherry-CAAX fusion tag (Addgene #50839). Stellar *E. coli* cells (TaKaRa) were transformed with these plasmids for amplification and DNA storage. All plasmids were sequenced by Genewiz to verify quality.

### Lentivirus production and transduction

HEK293T cells were plated on a 12-well plate, reaching 40% confluency the next day. The cells were then co-transfected with the corresponding pHR plasmid and lentiviral packaging plasmids (pMD and CMV) using Fugene HD (Promega). Cells were incubated for ~48 h and virus was collected and filtered through a 0.45-mm filter. In addition, 2 μL of polybrene and 40 μL of HEPES were added to the 2 mL viral solution. For infection, HEK293T cells were plated on a six-well plate, allowed to adhere, and reach 40% confluency. At that time, the cells were infected with 200–500 μL of viral solution. For iLID-SSPB translocation experiments, NIH3T3 cells were puromycin-selected after lentiviral transduction with an iLID-SSPB expression plasmid (pHR BFP-SSPB-SOScat-2A-PuroR-2A-iLID-CAAX) and a clonal cell line was established to limit cell-to-cell variability in expression. All imaging was done at least 48 h post infection. Cells were cultured in Dulbecco’s modified Eagle’s medium with 10% FBS, penicillin (100 U/mL), and streptomycin (0.1 mg/mL).

### Screening for light-responsive monobodies

A 6x-histidine tagged fusion of YFP and SH2 (His_6_-YFP-SH2) was grown in 500 mL of autoinduction^48^ media + Kanamycin (Kan) (50 μg/mL) for 16 h at 30 °C. Monobody HA4 and monobody-AsLOV2 chimeras were grown in 250 mL of autoinduction media + Kan for 16 h at 30 °C. For each test, monobody HA4 (used as control) and three different monobody-AsLOV2 chimeras were tested simultaneously. Cells were then harvested by centrifugation at 7500 × *g* for 20 min at 4 °C in a Lynx 4000 centrifuge (Sorvall™) and supernatant was discarded. Cell pellets were resuspended in Binding buffer (Tris 100 mM pH 8.0, NaCl 150 mM, glycerol 1%, and 5 mM imidazole) supplemented with 1 mM of PMSF, adding 8 mL to the cells containing His_6_-YFP-SH2 and 3 mL for the monobody HA4 and each of the chimeras. The resuspended cells were flash-frozen forming droplets directly into liquid nitrogen (LN2) and placed in small (LN2-cold) grinding vials to be disrupted using a CryoMill system (Spex sample prep®) with a cycle of 2 min grinding and 3 min cooling (14 times). The broken cell powder was thawed in 50 mL Falcon tubes at room temperature with the addition of 4 mL of Lysis buffer (Binding buffer supplemented with 1 mM PMSF and 2 mg/mL of DNAse) for His_6_-YFP-SH2 and 2 mL of Lysis buffer for monobody HA4 and each of the chimeras. Once thawed, the bacterial lysates were centrifuged at 25,000 × *g* in a Lynx 4000 centrifuge (Sorvall™) for 30 min at 4 °C and the supernatant (clarified lysate) was transferred to 15 mL conical tubes. To enhance AsLOV2 activity, we added FMN to a final concentration of 0.25 mg/mL, to each of the chimera-containing lysate, and then incubated for 15 min at 4 °C by vertical rotation in a tube Revolver/Rotator at 50 r.p.m. (Thermo Scientific™). The His_6_-YFP-SH2 supernatant was then mixed with 4.5 mL of a 50% suspension of Co-charged agarose resin (Talon®), previously equilibrated in Binding buffer, and incubated at 4 °C, rocking for 45 min. The suspension was sedimented by gravity and the supernatant discarded. The beads were then thoroughly washed with Binding buffer with approximately 50 times the resin or column volume. The beads (now with His_6_-YFP-SH2 bound) were then finally resuspended in Binding buffer to a total volume of 13.5 mL. The bead suspension was equally divided into nine conical tubes of 15 mL (having each 1.5 mL of the bead suspension). Four of these tubes were used for experiments under blue light with LED panels (450 nm and intensity of 45 μmol/m^2^/s) while the other four were used for experiments in the dark (wrapped in aluminum foil). Experiments were performed in a dark room and red light was used occasionally for visualization purposes. The remaining tube was left as a control for His_6_-YFP-SH2 binding. Samples of 2.5 mL of bacterial clarified lysates containing either the monobody HA4 supernatant or each one of the chimeras were added to separate tubes of resin. The mixtures were then incubated for 45 min (at 4 °C and constant vertical rotation of 20 r.p.m.) under blue light or dark conditions. Tubes were then allowed to settle at 4 °C under the same light conditions in which they were incubated, for 15–20 min. After carefully discarding the supernatants, the beads were washed 5–6 times: each time with 10 mL of Binding buffer and rotating at 20 r.p.m. for 15 min. After each wash, the mixtures were allowed to settle at 4 °C for 15–20 min under the same light conditions as they were incubated and the supernatants were again discarded. This step was repeated until beads were washed with approximately 40–50 resin volumes. During the course of the experiments, light conditions within the room were carefully held constant and red light was for visualization purposes used only when needed, specially to minimize any blue light exposure of the dark samples when opening the wrapped tubes to exchange buffer (washing, incubation, and elution). After the last wash, proteins were eluted with 2.5 mL of Elution buffer (Tris 100 mM pH 8.0, 150 mM NaCl, 1% glycerol, 500 mM Imidazole) and then equal volumes of elution samples (dark and light) for each chimera (and control) were loaded onto 12% polyacrylamide gels and resolved with SDS-PAGE. To calculate the difference in binding between light and dark conditions for each chimera, we performed densitometry calculations and used the integrated intensities of the bands corresponding to the chimeras applying the FIJI implementation of ImageJ^49^.

### Purification of binders and targets

HA4, monobody-AsLOV2 chimeras (OptoMB and variants), and YFP-SH2 constructs were purified using N-terminal 6x-histidine tag fusions and metal affinity chromatography. Chimeras were protected from excessive ambient light exposure to prevent potential protein destabilization and improve yields. This included covering the shakers with black blankets during expression, wrapping culture flasks and tubes (containing crude or purified proteins) with thick aluminum foil and performing the chromatography in the dark or with red light (when needed). OptoMB (and all its variants) were expressed at 18 °C for 3 days in 1 or 2 L of Autoinduction media^48^ (plus Kan 50 μg/mL). HA4 and YFP-SH2 were expressed in 1 or 2 L of Autoinduction media (plus Kan 50 μg/mL) for 16 h at 30 °C. Cells were then harvested by centrifugation at 7500 × *g* for 20 min at 4 °C in a Lynx 6000 centrifuge (Sorvall™) and supernatant was discarded. Each cell pellet was resuspended in between 8 and 12 mL of Binding buffer and frozen droplets were prepared as described above and immediately transferred to a large (LN2-cold) grinding tube. Cryogenic grinding was performed as described above. Broken cells (frozen powder) were thawed in 50 mL Falcon tubes at room temperature with the addition of Lysis buffer up to 5% of the initial cell culture volume. After clarifying the bacterial lysates by centrifugation as described above, these were loaded onto columns of 2–5 mL (50% suspension) of Co-charged resin (Talon®) previously equilibrated with Binding buffer (Tris 100 mM pH 8.0, NaCl 150 mM, glycerol 1%, and 5 mM Imidazole). Columns were washed with 40–50 column volumes of Binding buffer and proteins eluted with Elution buffer (Binding buffer supplemented with 250 mM of imidazole). Proteins were then run through SEC using a Hiprep™ HR 16/60 Sephacryl™ 200 with Buffer A (Tris 50 mM pH 8.0 and NaCl 150 mM) in an FPLC (ÄKTA pure from GE® Healthcare). The aliquots enriched with the target proteins were concentrated by centrifugation (in cycles of 10 min) at maximum speed in a Sorvall Legend XTR Benchtop centrifuge (Thermo Scientific™) using 15 mL centricons® (Millipore) with a cutoff selected according to the molecular size of each protein, until concentrations between 3 and 8 mg/mL were reached.

### OptoMB characterization by size exclusion chromatography

Purified monobody HA4 (8 μL from a 200 μM sample) or OptoMB_V416I_G528A_N538E_ fused to a SUMO tag (28 μL from a 42 μM sample) dissolved in Buffer A was mixed with YFP-SH2 (20 μL from a 58.8 μM sample in Buffer A) in approximately 1.2:1 molar ratio of binder to target and completed to a total volume of 100 μL of Buffer A. Each mixture was incubated for 10 min in either dark or blue light (450 nm) before loading the full volume (100 μL) onto a Superdex™ 200 16/300 column (GE® Healthcare), which was then run at 1 mL/min with Buffer A at 4 °C (using an ÄKTA pure from GE® Healthcare). Pure OptoMB_V416I_G528A_N538E_ samples (28 μL from a 42 μM sample) completed to 100 μL Buffer A were also loaded and run in either light condition. To test different light conditions, the column was either illuminated with wrapped blue LED strips (450 nm, with approximate intensity of 30 μmol/m^2^/s) or covered with aluminum foil for the total duration of the filtration (Supplementary Fig. 3a). For experiments in the dark, we also minimized light sources in the room and covered the chromatography cabinet with a black blanket. The SEC was monitored by UV absorbance at 280 nm.

### Intracellular imaging of HEK293T cells

For live cell imaging, we used 0.17 mm glass-bottomed black-walled 96-well plates (In Vitro Scientific). Glass was first treated with 10 μg/mL of fibronectin in PBS for 20 min. HEK293T cells expressing both SH2-mCherry-CAAX and either the monobody HA4 or OptoMB were then plated and allowed to adhere onto the plate before imaging. Mineral oil (50 μL) was added on top of each well with cells prior to imaging, to limit media evaporation. The mammalian cells were kept at 37 °C with 5% CO_2_ for the duration of the imaging experiments. The irFP and mCherry fluorescence were imaged using a Nikon Eclipse Ti microscope with a Prior linear motorized stage, a Yokogawa CSU-X1 spinning disk, an Agilent laser line module containing 405, 488, 561, and 650 nm lasers, an iXon DU897 EMCCD camera, and a ×40 oil immersion objective lens. An LED light source was used for photoexcitation with blue light (450 nm), which was delivered through a Polygon400 digital micro-mirror device (DMD; Mightex Systems). For all LED illumination experiments we adjusted the LED power to a final value of ~1 mW/cm^2^ at the sample plane, as measured by a MQ-510 Quantum light meter with separate sensor (Apogee Instruments) using an equivalent blue LED light source placed above the sample. To measure light-induced changes in cytosolic intensity, the background-subtracted mean fluorescence intensity was compared between timepoint 1 (before light) and timepoint 7 (after 2 min constant illumination).

### Imaging of coated agarose beads

Approximately 200 μL of Ni-NTA agarose slurry (50% suspension) (Qiagen) equilibrated in Buffer A were mixed with 500 μL of 100 μM of either monobody HA4, OptoMB, or OptoMB_V416L_ (AsLOV2 V416L variant) in an Eppendorf tube (covered with aluminum foil) and incubated by vertical rotation (at 20 r.p.m. for 20 min) at room temperature to allow binding through the 6x-histidine tag until saturation. The excess protein was then washed twice with 1 mL of Buffer A, centrifuging the beads at low speed for 1 min (1000 r.p.m. in a benchtop centrifuge) each time, finally discarding the excess of supernatant after the second wash. Then, 50 μL of a purified YFP-SH2 solution at 2 μM (with the 6x-histidine tag cleaved off) was added onto a 0.17-mm glass-bottomed black-walled 96-well plate (In Vitro Scientific) followed by 2 μL of the washed resin with the beads labeled with OptoMonobody (or monobody HA4 as control). The mixture was equilibrated for at least 1 h at room temperature and up to overnight at 4 °C prior to imaging, performed at room temperature. The same microscope setup as described for the cells imaging was used, except for the objective (×20 in this case), to follow YFP fluorescence over time on the surface of the bead. For the spatial control of the OptoMB-SH2 interaction on beads the same setup was used. Two beads in an area of around 200 × 250 μm were imaged at the same time applying a light (450 nm) mask, which uses a square ROI with a dimension of 120 × 120 μm to cover and illuminate only one bead. The YFP fluorescence was recorded over time (for a total of 1 h) for both, the illuminated and the unilluminated bead. Quantification was performed by measuring the change in YFP fluorescence intensity over time in a defined region on the surface of the bead (using ImageJ^49^) and subtracting the background.

### Calculation of binding kinetics by BLI

Measurements of the binding (*k*_on_) and unbinding (*k*_off_) rate constants, as well as the dissociation (or affinity) constant (*K*_d_) for HA4 monobody and OptoMB (including variants V416L and V416I-G528A-N538E) were performed on Octet RED96e instruments (ForteBio). Ni-NTA sensors (ForteBio) were first equilibrated using Buffer A for 10 min prior the measurement. A volume of 200 μL of Buffer A or protein solutions (previously dialyzed with Buffer A when needed) were added to clear 96-well plates. During the experimental run, the Ni-NTA sensors were first immersed in Buffer A to record the baseline. Protein binders were then loaded by switching to wells with solutions of 6x-histidine tagged HA4 monobody or OptoMB variants (with concentrations between 100 μg/mL and up to 1 mg/mL) until values of ~4 nm were reached (avoiding saturation of the sensors). The sensors were then transferred back into Buffer A to remove unbound protein. To measure the binding rate constant (*k*_on_) the sensors with bound monobody HA4 or OptoMB variants were subsequently shifted to wells containing various concentrations of YFP-SH2 (at concentrations indicated in Fig. 3a, b and Supplementary Fig. 2b). To measure the unbinding rate constant (*k*_off_), the sensors were then moved to wells containing Buffer A to trigger dissociation of YFP-SH2. To measure binding kinetics of the light state, the lid of the Octet remained open during the measurement and a blue LED panel was held above the 96-well plate, maintaining constant blue illumination for the duration of the experiment. For the OptoMB variant with the AsLOV2 mutations V416I-G528A-N538E, lit states were sufficiently long-lived to remain fully activated in response to pre-illumination with a blue light panel and the continuous illumination of the Octet sensors. The raw binding and unbinding data were simultaneously fit to models of the binding and unbinding reactions:

1 $$y_{{\mathrm{{bind}}}}^i\left( t \right) = \left[ {a_{\mathrm{{on}}}^i\left( {1 - {\mathop{\rm{e}}\nolimits} ^{ - \left( {k_{\mathrm{{on}}}\left[ {{\mathrm{SH2}}} \right]_i + k_{\mathrm{{off}}}} \right)t}} \right) + b_{\mathrm{{on}}}^i} \right]{\mathrm{e}}^{ - k_{\mathrm{{leak}}}t},$$

2 $$y_{\mathrm{{unbind}}}^i\left( t \right) = \left[ {a_{\mathrm{{off}}}^i{\mathop{\rm{e}}\nolimits} ^{ - k_{\mathrm{{off}}}t} + b_{\mathrm{{off}}}^i} \right]{\mathrm{e}}^{ - k_{\mathrm{{leak}}}t}.$$

This model incorporates the following dependent and independent variables: $y_{\mathrm{{bind}}}^i\left( t \right)$ refers to the *i*th binding curve; $y_{\mathrm{{unbind}}}^i\left( t \right)$ the *i*th unbinding curve; [SH2]_*i*_ refers to the concentration of YFP-SH2 used for the *i*th binding curve; and *t* is the time elapsed since the start of the binding/unbinding phase.

It also includes the following parameters: *k*_on_ is the on-rate (enforced to be identical across all binding and unbinding curves); *k*_off_ the off-rate (enforced to be identical across all binding and unbinding curves); *k*_leak_ represents the slow unbinding of His-tagged OptoMB from the probe, leading to a gradual decay of signal throughout the entire experiment; $a_{\mathrm{{on}}}^i$ the total change in signal due to SH2 binding for the *i*th curve; $b_{\mathrm{{on}}}^i$ the signal baseline during the binding phase for the *i*th curve; $a_{\mathrm{{off}}}^i$ the total change in signal due to SH2 unbinding for the *i*th curve; and $b_{\mathrm{{off}}}^i$ is the signal baseline during the unbinding phase for the *i*th curve.

The model thus contains 4 × *n* + 3 parameters, where *n* is the number of distinct SH2 concentrations tested. Fits were performed using nonlinear gradient descent using the MATLAB fmincon function. The MATLAB code used to perform the fits and parameter confidence interval calculations is available on Github at https://github.com/toettchlab/Gil2020.

### LCAC of SH2-tagged proteins using cobalt-immobilized OptoMB

To prepare our OptoMB resin and columns for LCAC, we incubated the purified variant OptoMB_V416I_G528A_N538E_ (having a SUMO tag in the N-terminus to boost expression, Supplementary Table 4) with Co-charged resin (Talon®) (50% suspension) previously equilibrated in Buffer A, in order to produce 2.5 mL of resin with approximately 8 mg of OptoMB bound per mL. The resin was rotated in 50 mL conical tubes (covered with aluminum foil) at room temperature for 20 min and then poured in 20 mL BioRad glass Econo-Columns and washed with Buffer A (50 column volumes). The use of imidazole in the Buffer A was avoided because it interferes with the photocycle of the AsLOV2 domain, enhancing the rate of recovery of the dark state thus reducing the lifetime of the lit state^50^, which would reduce the efficiency of light elution. Production of the OptoMB resin (and columns) and the LCAC procedure were carried out at room temperature within a carefully controlled dark room to avoid blue light contamination, using red light from LED panels occasionally for visualization purposes. Approximately 10–12 mL of clarified bacterial lysates (obtained as described before from 1 L cultures) containing YFP-SH2 or SH2-Pdc1p (without his-tags) were incubated with 2.5 mL of OptoMB resin in Buffer A, which was enough to saturate the OptoMB resin with binding targets. The mixture was then vertically rotated gently for 45 min in 15 mL conical tubes. The OptoMB resin was then poured into 10 mL BioRad glass Econo-Columns or left to settle to discard the supernatant by pipetting (experiments in batch). Approximately 80 column volumes of Buffer A were used to wash the OptoMB resin. When purifying in batch, 40 mL of Buffer A was added to the resin at a time in 50 mL conical tubes; after rotating for 5 min, the resin was left to settle to remove the supernatant by pipetting. This cycle was repeated until reaching 80 resin volumes. After washing, the resin was poured back into a 15 mL conical tube. When purifying in batch, for each light-elution step we added two times the resin volume of Buffer A and incubated with gentle rocking in front of a blue light (450 nm) LED panel (approximately 20 cm away and an intensity of 45 μmol/m^2^/s) for 10–15 min; then letting the resin settle while illuminated and recovering the light-eluted aliquot by pipetting. When purifying in columns, two column bed volumes of Buffer A were poured at a time while the column was exposed to blue light from three LED panels in a triangular conformation (surrounding the column), and collected by gravity. For both batch and column purifications, the elution steps were repeated until no more protein eluted from the resin (usually 4–5 times). In both methods, batch or column, after light elution we added four times the resin volume of Buffer A containing 250 mM of imidazole to elute the proteins still bound to the resin after light elution, needed to calculate yields and resin capacity. Samples from the different purification steps were resolved with SDS-PAGE, using 12% SDS-polyacrylamide gels.

### LCAC of SH2-tagged proteins using covalently coupled OptoMB

A total of 1–1.5 g (dry weight) of CNBr-activated Sepharose^TM^ 4B (GE® Healthcare) was resuspended in Conjugation buffer (NaCHO_3_ 0.1 M pH 8.3 and 500 mM NaCl) according to the manufacturer’s specifications. The excess of Conjugation buffer was discarded and the hydrated resin mixed with purified OptoMB_V416I_G528A_N538E_ (containing an N-terminal SUMO tag to boost expression, Supplementary Table 4), previously dialyzed against Conjugation buffer by centrifugation (using 15 mL Centricons® as describe above). For each preparation, 5 mL (50% suspension) of resin was conjugated with OptoMB at approximately 8 mg of OptoMB per mL of resin. The manufacturer’s protocol was followed with minor modifications, keeping all protein immobilization steps (conjugation, blockage, and acid/base washing cycles) in the dark by covering the tubes with thick aluminum foil. The acid/base washing cycles were reduced to one cycle, as the three washes recommended by the manufacturer results in loss of the FMN, the AsLOV2 co-factor (as evidenced by the loss of the protein’s yellow coloration immediately after the second wash). Once OptoMB was conjugated, the resin was washed and equilibrated with Buffer AS (Buffer A with salt concentration increased to 300 mM NaCl). The OptoMB-conjugated resins were used immediately or stored at 4 °C (protected from light with foil), where they are stable for up to at least a week (yielding same results as fresh resin). To purify YFP-SH2 or SH2-Pdc1p, approximately 10–15 mL of the clarified bacterial lysates obtained from 1 L cultures (as described above) were incubated in batch with 5 mL (50% suspension) of the OptoMB-conjugated sepharose resin, which was enough to saturate the OptoMB resin with binding targets. The same protocol for LCAC purification with OptoMB-immobilized in Co-charged (Talon®) in batch described above was followed with CNBr-OptoMB resin, except Buffer AS was used instead of Buffer A. To calculate the yields and resin capacity, after the final light-elution step, 200 μL of the 50% resin suspension were resuspended in 100 μL of SDS-PAGE sample-loading buffer and incubated at 100 °C for 10 min in a heat block, before loading onto the gel. Samples from all purification steps were resolved on a 12% SDS-polyacrylamide gels.

### Calculations of protein purity, yield and resin capacity

Protein purity was estimated using densitometric analysis in the FIJI implementation of ImageJ^49^. The software analyzes individual bands from a scanned gel, returning the integrated intensity of each band. For each aliquot of purified protein (eluted with light), we selected the band representing the SH2-tagged protein, and used their integrated intensities to calculate the fraction of the purified target versus the overall total of protein present in that fraction.

The overall yield (*y*) represents the total amount (mg) of purified protein that eluted with light (TPE) as a percentage of the total amount (mg) of protein that was initially bound to the resin (TPB). TPE was calculated by multiplying the concentration (mg/mL) of all the light-eluted fractions ([TPE]) by their total volume (mL) (TPEvol). The concentrations of the fractions eluted with light were calculated using absorbance at 280 nm with a spectrometer (Biospectrometer, Eppendorf). TPB was equal to TPE plus the total residual SH2-tagged protein remaining on the column after light elution (TRP). To calculate the TRP, we recovered all residual protein using imidazole elution (of Co-Agarose-OptoMB) or heat treatment (of CNBr-OptoMB) of the beads after light elution. Concentrations of recovered residual protein were calculated by running each sample on a 12% polyacrylamide gel alongside standards of the same SH2-tagged protein of known concentrations. Band intensities were then computed by densitometry analysis in FIJI as above. The amount of target protein still bound to the column was thus equal to the concentration retrieved in this final elution [TRP] multiplied by its total elution volume TRPvol. Finally, the resin capacity *C*_R_ was calculated as TPB divided by the total volume of the resin used *V*_R_.

3 $$y = \frac{{\mathrm{{TPE}} \times 100}}{{\mathrm{{TPB}}}},$$

4 $${\mathrm{{TPE}}} = \left[ {\mathrm{{TPE}}} \right] \times {\mathrm{{TPEvol}}},$$

5 $${\mathrm{{TPB}}} = \left\{ {\left[ {\mathrm{{TRP}}} \right] \times {\mathrm{{TRPvol}}}} \right\} + {\mathrm{{TPE}}},$$

6 $$\mathit{C}_{\mathrm{R}} = \frac{{\mathrm{{TPB}}}}{{V_{\mathrm{R}}}},$$

where *y* is the yield of purified protein obtained with the resin; TPE the total amount of protein eluted with light; [TPE] the concentration of all the fractions that eluted with light; TPEvol the total volume of the light-elution fraction; TPB the total amount of protein initially bound to the resin; [TRP] the concentration of the total residual protein fraction that was still bound to the resin after the elution with light; TRPvol is the total volume of the fraction containing the total residual protein that was still bound to the resin after the elution with light, and was recovered by imidazole or heat treatment of the beads; *C*_R_ the protein-binding capacity of the resin; and *V*_R_ is the volume of resin used in the experiment.

### Reporting summary

Further information on research design is available in the Nature Research Reporting Summary linked to this article.

## Supplementary information

Supplementary Information

Description of Additional Supplementary Files

Supplementary Movie 1

Supplementary Movie 2

Supplementary Movie 3

Supplementary Movie 4

Reporting Summary

## Data Availability

The authors declare that all data supporting the findings of this study are available within the paper (and its Supplementary Information files). The source data underlying Table 2, Figs. 1e, 2c, e, 3a–c, 5d, e, and Supplementary Figs. 2b, and 3b, c are provided in the Source Data File.

## Source data

Source Data

## Code availability

All microscopy data were collected using NIS Elements version 4.4 (Nikon), quantified in ImageJ v1.52p, and analyzed using custom MATLAB scripts, which are available on Github at https://github.com/toettchlab/Gil2020.
