# Supplementary material for: Development of light-responsive protein binding in the monobody non-immunoglobulin scaffold
Source: Nat Commun. 2020 Aug 13;11:4045. doi: 10.1038/s41467-020-17837-7 (PMC7427095; doi:10.1038/s41467-020-17837-7)
Supplement: Supplementary file 1 — Supplementary Information [file 41467_2020_17837_MOESM1_ESM.pdf]

Supplementary information to:

Development of light-responsive protein binding in the monobody non-immunoglobulin scaffold

César Carrasco-López[1,#], Evan M. Zhao[1,#], Agnieszka A. Gil[2,#], Nathan Alam[1], Jared E. Toettcher[2,*] and José. L. Avalos[1,3,*]

[1] Department of Chemical and Biological Engineering
Princeton University, Princeton NJ 08544

[2] Department of Molecular Biology
Princeton University, Princeton NJ 08544

[3] Andlinger Center for Energy and the Environment
Princeton University, Princeton NJ 08544

[#] These authors contributed to this work equally

* Co-corresponding Authors
toettcher@princeton.edu; javalos@princeton.edu

Supplementary Information

Supplementary Note 1

The AsLOV2 sequence is highlighted in blue.

HA4-AsLOV2 (SS58 insertion): αSH2-OptoMB
GSSVSSVPTKLEVVAATPTSLLISWDAPMSSSSVYYYRITYGETGGNSPVQEFTVPYSGLERIEKNFVITDPRLPDNPIIFASDSFLQLTEYSREEILGRNCRFLQGPETDRATVRKIRDAIDNQTEVTVQLINYTKSGKKFWNLFHLQPMRDQKGDVQYFIGVQLDGTEHVRDAAEREGVMLIKKTAENIDEAAGSSTATISGLSPGVDYTITVYAWGEDSAGYMFMYSPISINYRTC*

HA4-AsLOV2 (MS29 insertion with residues removed from Loop AB):
GSSVSSVPTKLEVVAATPTSLLISWDAPMGLERIEKNFVITDPRLPDNPIIFASDSFLQLTEYSREEILGRNCRFLQGPETDRATVRKIRDAIDNQTEVTVQLINYTKSGKKFWNLFHLQPMRDQKGDVQYFIGVQLDGTEHVRDAAEREGVMLIKKTAENIDEAAGSVYYYRITYGETGGNSPVQEFTVPYSSSTATISGLSPGVDYTITVYAWGEDSAGYMFMYSPISINYRTC*

HA4-AsLOV2 (MS29 insertion):
GSSVSSVPTKLEVVAATPTSLLISWDAPMGLERIEKNFVITDPRLPDNPIIFASDSFLQLTEYSREEILGRNCRFLQGPETDRATVRKIRDAIDNQTEVTVQLINYTKSGKKFWNLFHLQPMRDQKGDVQYFIGVQLDGTEHVRDAAEREGVMLIKKTAENIDEAAGSSSSVYYYRITYGETGGNSPVQEFTVPYSSSTATISGLSPGVDYTITVYAWGEDSAGYMFMYSPISINYRTC*

HA4-AsLOV2 (GN46 insertion with residues removed from Loop CD):
GSSVSSVPTKLEVVAATPTSLLISWDAPMSSSSVYYYRITYGETGGLERIEKNFVITDPRLPDNPIIFASDSFLQLTEYSREEILGRNCRFLQGPETDRATVRKIRDAIDNQTEVTVQLINYTKSGKKFWNLFHLQPMRDQKGDVQYFIGVQLDGTEHVRDAAEREGVMLIKKTAENIDEAAGNSPVQEFTVPYSSSTATISGLSPGVDYTITVYAWGEDSAGYMFMYSPISINYRTC*

HA4-AsLOV2 (TN45 insertion with residues removed from Loop CD):
GSSVSSVPTKLEVVAATPTSLLISWDAPMSSSSVYYYRITYGETGLERIEKNFVITDPRLPDNPIIFASDSFLQLTEYSREEILGRNCRFLQGPETDRATVRKIRDAIDNQTEVTVQLINYTKSGKKFWNLFHLQPMRDQKGDVQYFIGVQLDGTEHVRDAAEREGVMLIKKTAENIDEAAGNSPVQEFTVPYSSSTATISGLSPGVDYTITVYAWGEDSAGYMFMYSPISINYRTC*

HA4-AsLOV2 (SP68 insertion with residues removed from Loop EF):
GSSVSSVPTKLEVVAATPTSLLISWDAPMSSSSVYYYRITYGETGGNSPVQEFTVPYSSSTATISGSGLERIEKNFVITDPRLPDNPIIFASDSFLQLTEYSREEILGRNCRFLQGPETDRATVRKIRDAIDNQTEVTVQLINYTKSGKKFWNLFHLQPMRDQKGDVQYFIGVQLDGTEHVRDAAEREGVMLIKKTAENIDEAAGPVDYTITVYAWGEDSAGYMFMYSPISINYRTC*

HA4-AsLOV2 (PT18 insertion with residues removed from Loop AB):
GSSVSSVPTKLEVVAAPGLERIEKNFVITDPRLPDNPIIFASDSFLQLTEYSREEILGRNCRFLQGPETDRATVRKIRDAIDNQTEVTVQLINYTKSGKKFWNLFHLQPMRDQKGDVQYFIG

VQLDGTEHVRDAAEREGVMLIKKTAENIDEAAGTSLLISWDAPMSSSSVYYYRITYGET
GGNSPVQEFTVPYSSSTATISGLSPGVDYTITVYAWGEDSAGYMFMYSPISINYRTC*

AsLOV2 domain:
GLERIEKNFVITDPRLPDNPIIFASDSFLQLTEYSREEILGRNCRFLQGPETDRATVRKIRD
AIDNQTEVTVQLINYTKSGKKFWNLFHLQPMRDQKGDVQYFIGVQLDGTEHVRDAAER
EGVMLIKKTAENIDEAAG

Monobody HA4:
GSSVSSVPTKLEVVAATPTSLLISWDAPMSSSSVYYYRITYGETGGNSPVQEFTVPYSSST
ATISGLSPGVDYTITVYAWGEDSAGYMFMYSPISINYRTC*

His_6-YFP-SH2:
HHHHHHSSGENLYFQGHMASKVSKGEELFTGVVPILVELDGDVNGHKFSVSGEGEGDA
TYGKLTLKFICTTGKLPVPWPTLVTTFGYGLQCFARYPDHMKQHDFFKSAMPEGYVQE
RTIFFKDDGNYKTRAEVKFEGDTLVNRIELKGIDFKEDGNILGHKLEYNYNSHNVYIMA
DKQKNGIKVNFKIRHNIEDGSVQLADHYQQNTPIGDGPVLLPDNHYLSTQSALSKDPNE
KRDHMVLLEFVTAAGITLGMDELYKSLEKHSWYHGPVSRNAAEYLLSSGINGSFLVRES
ESSPGQRSISLRYEGRVYHYRINTASDGKLYVSSESRFNTLAELVHHHSTVADGLITTLH
YPAPKRNKPTVYGVSPNY*

HA4-AsLOV2 (SS30 insertion):
GSSVSSVPTKLEVVAATPTSLLISWDAPMSGLERIEKNFVITDPRLPDNPIIFASDSFLQLT
EYSREEILGRNCRFLQGPETDRATVRKIRDAIDNQTEVTVQLINYTKSGKKFWNLFHLQP
MRDQKGDVQYFIGVQLDGTEHVRDAAEREGVMLIKKTAENIDEAAGSSSVYYYRITYG
ETGGNSPVQEFTVPYSSSTATISGLSPGVDYTITVYAWGEDSAGYMFMYSPISINYRTC*

HA4-AsLOV2 (NS47 insertion):
GSSVSSVPTKLEVVAATPTSLLISWDAPMSSSSVYYYRITYGETGGNGLERIEKNFVITDP
RLPDNPIIFASDSFLQLTEYSREEILGRNCRFLQGPETDRATVRKIRDAIDNQTEVTVQLIN
YTKSGKKFWNLFHLQPMRDQKGDVQYFIGVQLDGTEHVRDAAEREGVMLIKKTAENI
DEAAGSPVQEFTVPYSSSTATISGLSPGVDYTITVYAWGEDSAGYMFMYSPISINYRTC*

HA4-AsLOV2 (SA84 insertion):
GSSVSSVPTKLEVVAATPTSLLISWDAPMSSSSVYYYRITYGETGGNSPVQEFTVPYSSST
ATISGLSPGVDYTITVYAWGEDSGLERIEKNFVITDPRLPDNPIIFASDSFLQLTEYSREEIL
GRNCRFLQGPETDRATVRKIRDAIDNQTEVTVQLINYTKSGKKFWNLFHLQPMRDQKG
DVQYFIGVQLDGTEHVRDAAEREGVMLIKKTAENIDEAAGAGYMFMYSPISINYRTC*

YFP-SH2:
MASKVSKGEELFTGVVPILVELDGDVNGHKFSVSGEGEGDATYGKLTLKFICTTGKLPV
PWPTLVTTFGYGLQCFARYPDHMKQHDFFKSAMPEGYVQERTIFFKDDGNYKTRAEVK
FEGDTLVNRIELKGIDFKEDGNILGHKLEYNYNSHNVYIMADKQKNGIKVNFKIRHNIED
GSVQLADHYQQNTPIGDGPVLLPDNHYLSTQSALSKDPNEKRDHMVLLEFVTAAGITLG
MDELYKSLEKHSWYHGPVSRNAAEYLLSSGINGSFLVRESESSPGQRSISLRYEGRVYH

YRINTASDGKLYVSSESRFNTLAELVHHHSTVADGLITTLHYPAPKRNKPTVYGVSPNY*

His$_6$-HA4:
HHHHHHSSGENLYFQGHASGSSVSSVPTKLEVVAATPTSLLISWDAPMSSSSVYYYRITYGETGGNSPVQEFTVPYSSSTATISGLSPGVDYTITVYAWGEDSAGYMFMYSPISINYRTC*

HA4-AsLOV2 (TG44 insertion):
GSSVSSVPTKLEVVAATPTSLLISWDAPMSSSSVYYYRITYGETGLERIEKNFVITDPRLPDNPIIFASDSFLQLTEYSREEILGRNCRFLQGPETDRATVRKIRDAIDNQTEVTVQLINYTKSGKKFWNLFHLQPMRDQKGDVQYFIGVQLDGTEHVRDAAEREGVMLIKKTAENIDEAAGGGNSPVQEFTVPYSSSTATISGLSPGVDYTITVYAWGEDSAGYMFMYSPISINYRTC*

HA4-AsLOV2 (SG65 insertion):
GSSVSSVPTKLEVVAATPTSLLISWDAPMSSSSVYYYRITYGETGGNSPVQEFTVPYSSSTATISGLERIEKNFVITDPRLPDNPIIFASDSFLQLTEYSREEILGRNCRFLQGPETDRATVRKIRDAIDNQTEVTVQLINYTKSGKKFWNLFHLQPMRDQKGDVQYFIGVQLDGTEHVRDAAEREGVMLIKKTAENIDEAAGGLSPGVDYTITVYAWGEDSAGYMFMYSPISINYRTC*

HA4-AsLOV2 (DA26 insertion):
GSSVSSVPTKLEVVAATPTSLLISWDGLERIEKNFVITDPRLPDNPIIFASDSFLQLTEYSREEILGRNCRFLQGPETDRATVRKIRDAIDNQTEVTVQLINYTKSGKKFWNLFHLQPMRDQKGDVQYFIGVQLDGTEHVRDAAEREGVMLIKKTAENIDEAAGAPMSSSSVYYYRITYGETGGNSPVQEFTVPYSSSTATISGLSPGVDYTITVYAWGEDSAGYMFMYSPISINYRTC*

HA4-AsLOV2 (ED82 insertion):
GSSVSSVPTKLEVVAATPTSLLISWDAPMSSSSVYYYRITYGETGGNSPVQEFTVPYSSSTATISGLSPGVDYTITVYAWGEGLERIEKNFVITDPRLPDNPIIFASDSFLQLTEYSREEILGRNCRFLQGPETDRATVRKIRDAIDNQTEVTVQLINYTKSGKKFWNLFHLQPMRDQKGDVQYFIGVQLDGTEHVRDAAEREGVMLIKKTAENIDEAAGDSAGYMFMYSPISINYRTC*

HA4-AsLOV2 (DS83 insertion):
GSSVSSVPTKLEVVAATPTSLLISWDAPMSSSSVYYYRITYGETGGNSPVQEFTVPYSSSTATISGLSPGVDYTITVYAWGEDGLERIEKNFVITDPRLPDNPIIFASDSFLQLTEYSREEILGRNCRFLQGPETDRATVRKIRDAIDNQTEVTVQLINYTKSGKKFWNLFHLQPMRDQKGDVQYFIGVQLDGTEHVRDAAEREGVMLIKKTAENIDEAAGSAGYMFMYSPISINYRTC*

HA4-AsLOV2 (GG45 insertion):
GSSVSSVPTKLEVVAATPTSLLISWDAPMSSSSVYYYRITYGETGGLERIEKNFVITDPRLPDNPIIFASDSFLQLTEYSREEILGRNCRFLQGPETDRATVRKIRDAIDNQTEVTVQLINYTKSGKKFWNLFHLQPMRDQKGDVQYFIGVQLDGTEHVRDAAEREGVMLIKKTAENIDEAAGGNSPVQEFTVPYSSSTATISGLSPGVDYTITVYAWGEDSAGYMFMYSPISINYRTC*

HA4-AsLOV2 (PT18 insertion):
GSSVSSVPTKLEVVAATPGLERIEKNFVITDPRLPDNPIIFASDSFLQLTEYSREEILGRNCR

FLQGPETDRATVRKIRDAIDNQTEVTVQLINYTKSGKKFWNLFHLQPMRDQKGDVQYFIGVQLDGTEHVRDAAEREGVMLIKKTAENIDEAAGTSLLISWDAPMSSSSVYYYRITYGETGGNSPVQEFTVPYSSSTATISGLSPGVDYTITVYAWGEDSAGYMFMYSPISINYRTC*

HA4-AsLOV2 (SP68 insertion):
GSSVSSVPTKLEVVAATPTSLLISWDAPMSSSSVYYYRITYGETGGNSPVQEFTVPYSSSTATISGLSGLERIEKNFVITDPRLPDNPIIFASDSFLQLTEYSREEILGRNCRFLQGPETDRATVRKIRDAIDNQTEVTVQLINYTKSGKKFWNLFHLQPMRDQKGDVQYFIGVQLDGTEHVRDAAEREGVMLIKKTAENIDEAAGPGVDYTITVYAWGEDSAGYMFMYSPISINYRTC*

AsLOV2-HA4 (N-terminus):
GLERIEKNFVITDPRLPDNPIIFASDSFLQLTEYSREEILGRNCRFLQGPETDRATVRKIRDAIDNQTEVTVQLINYTKSGKKFWNLFHLQPMRDQKGDVQYFIGVQLDGTEHVRDAAEREGVMLIKKTAENIDEAAGGSSVSSVPTKLEVVAATPTSLLISWDAPMSSSSVYYYRITYGETGGNSPVQEFTVPYSSSTATISGLSPGVDYTITVYAWGEDSAGYMFMYSPISINYRTC*

AsLOV2-HA4 (C-terminus):
GSSVSSVPTKLEVVAATPTSLLISWDAPMSSSSVYYYRITYGETGGNSPVQEFTVPYSSSTATISGLSPGVDYTITVYAWGEDSAGYMFMYSPISINYRTCGLERIEKNFVITDPRLPDNPIIFASDSFLQLTEYSREEILGRNCRFLQGPETDRATVRKIRDAIDNQTEVTVQLINYTKSGKKFWNLFHLQPMRDQKGDVQYFIGVQLDGTEHVRDAAEREGVMLIKKTAENIDEAAG*

HA4-AsLOV2 (SS59 insertion):
GSSVSSVPTKLEVVAATPTSLLISWDAPMSSSSVYYYRITYGETGGNSPVQEFTVPYSSGLERIEKNFVITDPRLPDNPIIFASDSFLQLTEYSREEILGRNCRFLQGPETDRATVRKIRDAIDNQTEVTVQLINYTKSGKKFWNLFHLQPMRDQKGDVQYFIGVQLDGTEHVRDAAEREGVMLIKKTAENIDEAAGSTATISGLSPGVDYTITVYAWGEDSAGYMFMYSPISINYRTC*

HA4-AsLOV2 (MY90 insertion):
GSSVSSVPTKLEVVAATPTSLLISWDAPMSSSSVYYYRITYGETGGNSPVQEFTVPYSSSTATISGLSPGVDYTITVYAWGEDSAGYMFMGLERIEKNFVITDPRLPDNPIIFASDSFLQLTEYSREEILGRNCRFLQGPETDRATVRKIRDAIDNQTEVTVQLINYTKSGKKFWNLFHLQPMRDQKGDVQYFIGVQLDGTEHVRDAAEREGVMLIKKTAENIDEAAGYSPISINYRTC*

His_6-HA4-AsLOV2 (SS58 insertion):
HHHHHHSSGENLYFQGHASGSSVSSVPTKLEVVAATPTSLLISWDAPMSSSSVYYYRITYGETGGNSPVQEFTVPYSGLERIEKNFVITDPRLPDNPIIFASDSFLQLTEYSREEILGRNCRFLQGPETDRATVRKIRDAIDNQTEVTVQLINYTKSGKKFWNLFHLQPMRDQKGDVQYFIGVQLDGTEHVRDAAEREGVMLIKKTAENIDEAAGSSTATISGLSPGVDYTITVYAWGEDSAGYMFMYSPISINYRTC*

HA4-AsLOV2 (YS57 insertion):
GSSVSSVPTKLEVVAATPTSLLISWDAPMSSSSVYYYRITYGETGGNSPVQEFTVPYGLERIEKNFVITDPRLPDNPIIFASDSFLQLTEYSREEILGRNCRFLQGPETDRATVRKIRDAIDNQTEVTVQLINYTKSGKKFWNLFHLQPMRDQKGDVQYFIGVQLDGTEHVRDAAEREGVMLIKKTAENIDEAAGSSSTATISGLSPGVDYTITVYAWGEDSAGYMFMYSPISINYRTC*

His$_6$-HA4-AsLOV2 (SS58 insertion, C450V mutant):
HHHHHHSSGENLYFQGHASGSSVSSVPTKLEVVAATPTSLLISWDAPMSSSSVYYYRITY
GETGGNSPVQEFTVPYSGLERIEKNFVITDPRLPDNPIIFASDSFLQLTEYSREEILGRNVR
FLQGPETDRATVRKIRDAIDNQTEVTVQLINYTKSGKKFWNLFHLQPMRDQKGDVQYFI
GVQLDGTEHVRDAAEREGVMLIKKTAENIDEAAGSSTATISGLSPGVDYTITVYAWGED
SAGYMFMYSPISINYRTC*

SH2-PDC1:
MGSLEKHSWYHGPVSRNAAEYLLSSGINGSFLVRESESSPGQRSISLRYEGRVYHYRINT
ASDGKLYVSSESRFNTLAELVHHHSTVADGLITTLHYPAPKRNKPTVYGVSPNYASSEIT
LGKYLFERLKQVNVNTVFGLPGDFNLSLLDKIYEVEGMRWAGNANELNAAYAADGYA
RIKGMSCIITTFGVGELSALNGIAGSYAEHVGVLHVVGVPSISAQAKQLLLHHTLGNGDF
TVFHRMSANISETTAMITDIATAPAEIDRCIRTTYVTQRPVYLGLPANLVDLNVPAKLLQ
TPIDMSLKPNDAESEKEVIDTILALVKDAKNPVILADACCSRHDVKAETKKLIDLTQFPA
FVTPMGKGSIDEQHPRYGGVYVGTLSKPEVKEAVESADLILSVGALLSDFNTGSFSYSY
KTKNIVEFHSDHMKIRNATFPGVQMKFVLQKLLTTIADAAKGYKPVAVPARTPANAAV
PASTPLKQEWMWNQLGNFLQEGDVVIAETGTSAFGINQTTFPNNTYGISQVLWGSIGFT
TGATLGAAFAAEEIDPKKRVILFIGDGSLQLTVQEISTMIRWGLKPYLFVLNNDGYTIEKL
IHGPKAQYNEIQGWDHLSLLPTFGAKDYETHRVATTGEWDKLTQDKSFNDNSKIRMIEI
MLPVFDAPQNLVEQAKLTAATNAKQ*

His$_6$-SUMO-HA4-AsLOV2 (SS58 insertion, V416I, G528A, N538E mutant):
HHHHHHGSGSGSDQEAKPSTEDLGDKKEGEYIKLKVIGQDSSEIHFKVKMTTHLKKLKE
SYCQRQGVPMNSLRFLFEGQRIADNHTPKELGMEEEDVIEVYQEQTGGHMASKGSSVSS
VPTKLEVVAATPTSLLISWDAPMSSSSVYYYRITYGETGGNSPVQEFTVPYSGLERIEKN
FIITDPRLPDNPIIFASDSFLQLTEYSREEILGRNCRFLQGPETDRATVRKIRDAIDNQTEVT
VQLINYTKSGKKFWNLFHLQPMRDQKGDVQYFIGVQLDGTEHVRDAAEREAVMLIKK
TAEEIDEAAGSSTATISGLSPGVDYTITVYAWGEDSAGYMFMYSPISINYRTC*

His$_6$-HA4-AsLOV2 (SS58 insertion, V416I mutant):
HHHHHHSSGENLYFQGHASGSSVSSVPTKLEVVAATPTSLLISWDAPMSSSSVYYYRITY
GETGGNSPVQEFTVPYSGLERIEKNFIITDPRLPDNPIIFASDSFLQLTEYSREEILGRNCRF
LQGPETDRATVRKIRDAIDNQTEVTVQLINYTKSGKKFWNLFHLQPMRDQKGDVQYFIG
VQLDGTEHVRDAAEREGVMLIKKTAENIDEAAGSSTATISGLSPGVDYTITVYAWGEDS
AGYMFMYSPISINYRTC*

HA4-irFP
MGSSVSSVPTKLEVVAATPTSLLISWDAPMSSSSVYYYRITYGETGGNSPVQEFTVPYSS
STATISGLSPGVDYTITVYAWGEDSAGYMFMYSPISINYRTCGGGAEGSVARQPDLLTC
DDEPIHIPGAIQPHGLLLALAADMTIVAGSDNLPELTGLAIGALIGRSAADVFDSETHNRL
TIALAEPGAAVGAPITVGFTMRKDAGFIGSWHRHDQLIFLELEPPQRDVAEPQAFFRRTN
SAIRRLQAAETLESACAAAAQEVRKITGFDRVMIYRFASDFSGEVIAEDRCAEVESKLGL
HYPASTVPAQARRLYTINPVRIIPDINYRPVPVTPDLNPVTGRPIDLSFAILRSVSPVHLEF
MRNIGMHGTMSISILRGERLWGLIVCHHRTPYYVDLDGRQACELVAQVLAWQIGVMEE
AAATPTCNMRD*

HA4-AsLOV2-irFP (SS58 insertion)

MGSSVSSVPTKLEVVAATPTSLLISWDAPMSSSSVYYYRITYGETGGNSPVQEFTVPYSGLERIEKNFVITDPRLPDNPIIFASDSFLQLTEYSREEILGRNCRFLQGPETDRATVRKIRDAIDNQTEVTVQLINYTKSGKKFWNLFHLQPMRDQKGDVQYFIGVQLDGTEHVRDAAEREGVMLIKKTAENIDEAAGSSTATISGLSPGVDYTITVYAWGEDSAGYMFMYSPISINYRTCGGGAEGSVARQPDLLTCDDEPIHIPGAIQPHGLLLALAADMTIVAGSDNLPELTGLAIGALIGRSAADVFDSETHNRLTIALAEPGAAVGAPITVGFTMRKDAGFIGSWHRHDQLIFLELEPPQRDVAEPQAFFRRTNSAIRRLQAAETLESACAAAAQEVRKITGFDRVMIYRFASDFSGEVIAEDRCAEVESKLGLHYPASTVPAQARRLYTINPVRIIPDINYRPVPVTPDLNPVTGRPIDLSFAILRSVSPVHLEFMRNIGMHGTMSISILRGERLWGLIVCHHRTPYYVDLDGRQACELVAQVLAWQIGVMEEAAATPTCNMRD*

SH2-mCherry-CAAX

MSLEKHSWYHGPVSRNAAEYLLSSGINGSFLVRESESSPGQRSISLRYEGRVYHYRINTASDGKLYVSSESRFNTLAELVHHHSTVADGLITTLHYPAPKRNKPTVYGVSPNYVSKGEEDNMAIIKEFMRFKVHMEGSVNGHEFEIEGEGEGRPYEGTQTAKLKVTKGGPLPFAWDILSPQFMYGSKAYVKHPADIPDYLKLSFPEGFKWERVMNFEDGGVVTVTQDSSLQDGEFIYKVKLRGTNFPSDGPVMQKKTMGWEASSERMYPEDGALKGEIKQRLKLKDGGHYDAEVKTTYKAKKPVQLPGAYNVNIKLDITSHNEDYTIVEQYERAEGRHSTGGMDELYKGSGSGSKKKKKKSKTKCVIM

Supplementary Note 2

We performed a search for monobodies in the Protein Data Bank (PDB), using “monobody” as key word. Our search resulted in a total of 51 different structures of monobodies, each one in complex with their cognate target. From this total, only 32 were monobodies with unique protein sequences, which are included in Supplementary Table 3. A close inspection of the mode of binding of each unique monobody in complex with its target revealed that 23 (72%) of them bind their cognate targets in a side-binding mode, including monobody HA4 (see Supplementary Figure 6). The side-binding mode involves the Loop FG, one or more of the β-strands from βS2 and, occasionally, the CD loop.

Supplementary Tables

Supplementary Table 1. List of HA4-AsLOV2 chimeras showing insertion positions.

Chimera Name	Positions in HA4 where AsLOV2 was inserted
SS30	Inserted between S30 and S31
NS47	Inserted between N47 and S48
SA84	Inserted between S84 and A85
TG44	Inserted between T44 and G45
SS58	Inserted between S58 and S59
SG65	Inserted between S65 and G66
DA26	Inserted between D26 and A27
MS29	Inserted between M29 and S30
MS29-3	Inserted between M29 and S33 residues 30-32 were removed
ED82	Inserted between E82 and D83
DS83	Inserted between D83 and S84
GG45	Inserted between G45 and G46
GN45	Inserted between G45 and N48 residues 46-47 were removed
TN44	Inserted between T44 and N48 residues 45-47 were removed
PT18	Inserted between P18 and T19
SP68	Inserted between S68 and P69
SP68-1	Inserted between S68 and P69 residue 67 was removed
N-terminus	Inserted at N-terminus
C-terminus	Inserted at C-terminus
SS59	Inserted between S59 and S60
PT18	Inserted between P18 and T19 residue 17 was removed
MY90	Inserted between M90 and Y91
YS57	Inserted between Y57 and S58

Supplementary Table 2. Rate and dissociation constants from all individual BLI experiments.

Variant	State measured	Illumination	k_{on} (μM^{-1} s^{-1})	k_{off} (s^{-1})	K_d (μM)
Monobody HA4	-	ambient	0.0631	0.0145	0.23
OptoMB 1	Lit conformation	ambient*	<0.001	0.34	>100
OptoMB 2	Lit conformation	450 nm light	<0.001	0.21	>100
OptoMB$_{C450V}$ **1**	Dark conformation	ambient**	0.11	0.006	0.06
OptoMB$_{C450V}$ 2	Dark conformation	ambient**	0.056	0.013	0.23
OptoMB$_{C450V}$ 3	Dark conformation	450 nm light	0.048	0.013	0.27
OptoMB$_{V416L}$ 1	Lit conformation	ambient*	0.0029	0.25	87
OptoMB$_{V416L}$ 2	Lit conformation	450 nm light	0.0033	0.20	60
OptoMB$_{V416L}$ 3	Lit conformation	450 nm light	0.0058	0.24	42
#OptoMB$_{V416I_G528A_N538E}$ 1	Lit conformation	ambient*	<0.001	0.10	>100
#OptoMB$_{V416I_G528A_N538E}$ 2	Lit conformation	ambient*	<0.001	0.13	>100

*Due to the sensitivity of these constructs, ambient light in the laboratory as well as internal light from the digital panels of the Octet (BLI instrument) was sufficient to trigger the lit conformation.**This is a light-insensitive mutant and neither ambient nor LED light affected its basal dark conformation.

#SUMO tagged

Supplementary Table 3. Structurally characterized monobodies and their target binding mode (source: PDB).

PDB code	Monobody	Target	Binding Mode	Library*	Ref.
2OCF	E2#23	Human estrogen receptor	Side Binding	FG-Loop (FG7)	Koide *et al.*, 2002[1]
3K2M	HA4	Abl1 SH2 domain	Side Binding	Loops-only	Wojcik *et al.*, 2010[2]
3QHT	ySMB-1	Yeast SUMO	Side Binding	Loops-only	Gilbreth *et al.*, 2011[3]
3RZW	ySMB-9	Human SUMO	Loops Binding	Loops-only	Koide *et al.*, 2012[4]
3T04	7c12	Abl1 SH2 domain	Side Binding	Loops-only	Grebien *et al.*, 2011[5]
3UYO	SH13	Abl1 SH2 domain	Side Binding	Side and Loop	Koide *et al.*, 2012[4]
4HUK	MbMATE1	MATE multidrug transporter	Side Binding	Loops-only	Lu *et al.*, 2013[6]
4JE4	NSa1	NHP2 N-SH2 domain	Side Binding	Side and Loop	Sha *et al.*, 2013[7]
4JEG	CS1	NHP2 C-SH2 domain	Side Binding	Side and Loop	Sha *et al.*, 2013[7]
5A40	Bpe-S7	Fluoride Ion channel	Side Binding	Side and Loop	Stockbridge *et al.*, 2014[8] and 2015[9]
5A43	Bpe-L2	Fluoride Ion channel	Loops Binding	Loops-only	Stockbridge *et al.*, 2014[8] and 2015[9]
5DC0	GG3	Abl1 SH2 domain	Side Binding	Side and Loop	Wojcik *et al.*, 2016[10]
5DC4	AS25	Abl1 SH2 domain	Side Binding	Side and Loop	Wojcik *et al.*, 2016[10]
5E95	NS1	H-RAS	Side Binding	Side and Loop	Spencer-Smith *et al.*, 2017[11]
5ECJ	S4	Prdm14	Side Binding	Not specified	Nady *et al.*, 2015[12]
5G15	Mb1	Aurora A	Loops Binding	Not specified	Zorba *et al.*, 2019[13]
5KBN	S9	Fluoride channel F80I Mutant	Loops Binding	Side and Loop	Last *et al.*, 2016[14]
5KVM	α5	GPR56 ERC	Side Binding	Side and Loop	Salzman *et al.*, 2016[15]
5MTJ	Yes_1	Yes1-SH2	Side Binding	Side and Loop	Kükenshöner *et al.*, 2017[16]
5MTM	Lck_3	Lck-SH2	Side Binding	Side and Loop	Kükenshöner *et al.*, 2017[16]
5MTN	Lck_3	Lck-SH2	Side Binding	Side and Loop	Kükenshöner *et al.*, 2017[16]
5N7E	Bcr-DH_4	Dbl homology domain of Bcr-Abl	Side Binding	Side and Loop	Reckel *et al.*, 2017[17]
5NKQ	Ec2-S9	Fluoride Ion channel	Side Binding	Side and Loop	Stockbridge *et al.*, 2015[9]
5OC7	Bcr-PH_4	Pleckstrin-homology domain of Bcr-Abl	Side Binding	Not specified	Reckel *et al.*, 2017[17]

5V7P	MbRas1	Ras Methyltransferase ICMT	Loops Binding	Not specified	Diver *et al*., 2018[18]
6APX	YSX1	Human phosphatase 1 catalytic domain	Loops Binding	Loops-only	Gumpena *et al*., 2017[19]
6BQO	Bpe-S8	Fluoride Ion channel	Side Binding	Side and Loop	Stockbridge *et al*., 2014[8] and McIlwain *et al*., 2017[20]
6BX4	S9m	Fluoride Ion channel	Loops Binding	Side and Loop	Turman *et al*., 2018[21]
6BX5	S12	Fluoride Ion channel	Loops Binding	Side and Loop	Turman *et al*., 2018[21]
6BYN	S4	WDR5	Side Binding	Side and Loop	Gupta et al., 2018[22]
6C83	Mb2	Aurora A	Loops Binding	Not specified	Zorba *et al*., 2019[13]
6D0J	X1	CLC-Fluoride proton antiporter	Side Binding	Not specified	Last *et al*., 2018[23]

*Loops-only library diversified Loops BC, DE and FG; the Side and Loop libraries have at least two versions reported, one includes diversification of FG loop and several positions in βS2; another library diversifies the Loop CD in addition to FG and the positions in βS2; the FG-Loop library diversifies only the Loop FG.

Supplementary Table 4. Constructs used in this study.

Plasmid	Tag	Protein	Marker	Vector type
EZ-L663	None	HA4	Kanamycin	pCri-7b
EZ-L664	His$_6$	YFP-SH2	Kanamycin	pCri-8b
EZ-L703	None	YFP-SH2	Kanamycin	pCri-7b
EZ-L704	His$_6$	HA4	Kanamycin	pCri-8b
EZ-L706	None	HA4-AsLOV2 (SS58 insertion)	Kanamycin	pCri-7b
EZ-L736	None	HA4-AsLOV2 (MS29 insertion, residues 30-32 were removed)	Kanamycin	pCri-7b
EZ-L747	None	HA4-AsLOV2 (SS59 insertion)	Kanamycin	pCri-7b
EZ-L765	His$_6$	HA4-AsLOV2 (SS58 insertion)	Kanamycin	pCri-8b
EZ-L830	His$_6$-SUMO	HA4-AsLOV2 (SS58 insertion, V416L mutant)	Kanamycin	pCri-11b
EZ-L884	His$_6$	HA4-AsLOV2 (SS58 insertion, C450V mutant)	Kanamycin	pCri-8b
EZ-L886	None	SH2-PDC1	Kanamycin	pCri-7b
EZ-L889	His$_6$-SUMO	HA4-AsLOV2 (SS58 insertion, V416I, G528A, N538E mutant)	Kanamycin	pCri-11b
EZ-L892	His$_6$	HA4-AsLOV2 (SS58 insertion, V416I mutant)	Kanamycin	pCri-8b
AG-pHR1	None	HA4-iRFP	Ampicillin	pHR
AG-pHR2	None	HA4-AsLOV2-iRFP (SS58 insertion)	Ampicillin	pHR
AG-pHR3	CAAX	SH2-mCherry-CAAX	Ampicillin	pHR

Supplementary Figures

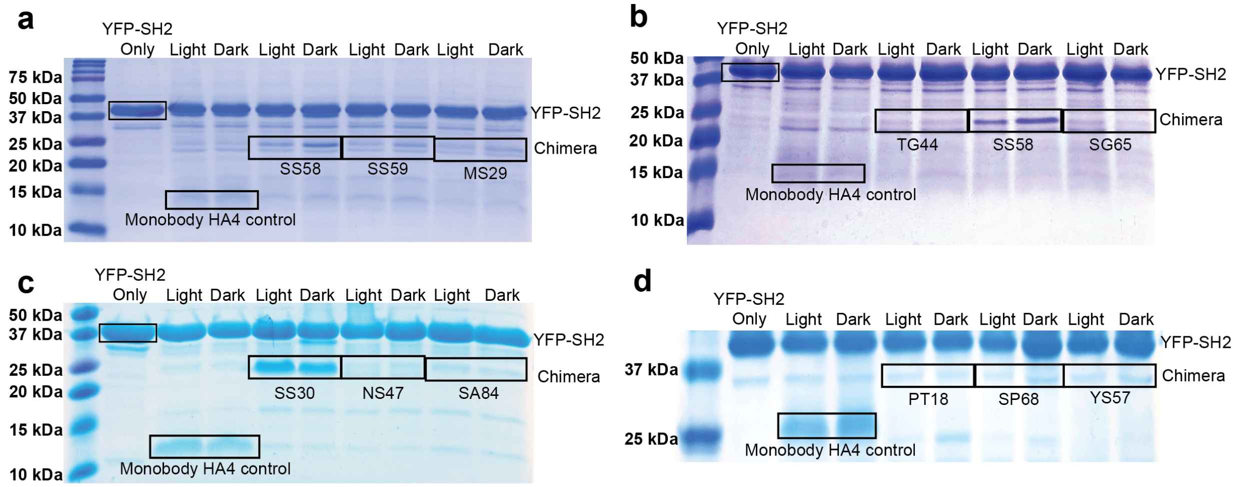

Supplementary Fig. 1: Representative SDS-PAGE gels of pull-down screens of HA4-AsLOV2 chimeras. a, Complete SDS-PAGE gel of the results shown in Fig. 1d, including chimeras with the AsLOV2 domain inserted in positions SS58, SS59 and MS29 of HA4 (with residues S30 to S32 of Loop BC deleted). **b-d**, A representative sample of SDS-PAGE gels of other chimeras with the AsLOV2 domain inserted in different positions that either do not bind or bind poorly to SH2 (TG44, NS47, SA84, YS57 and PT18) including at two positions in Loop EF (SG65, SP68); or bind well to SH2, but show no difference in binding between light conditions (SS30). All pull-downs were repeated at least twice observing similar results.

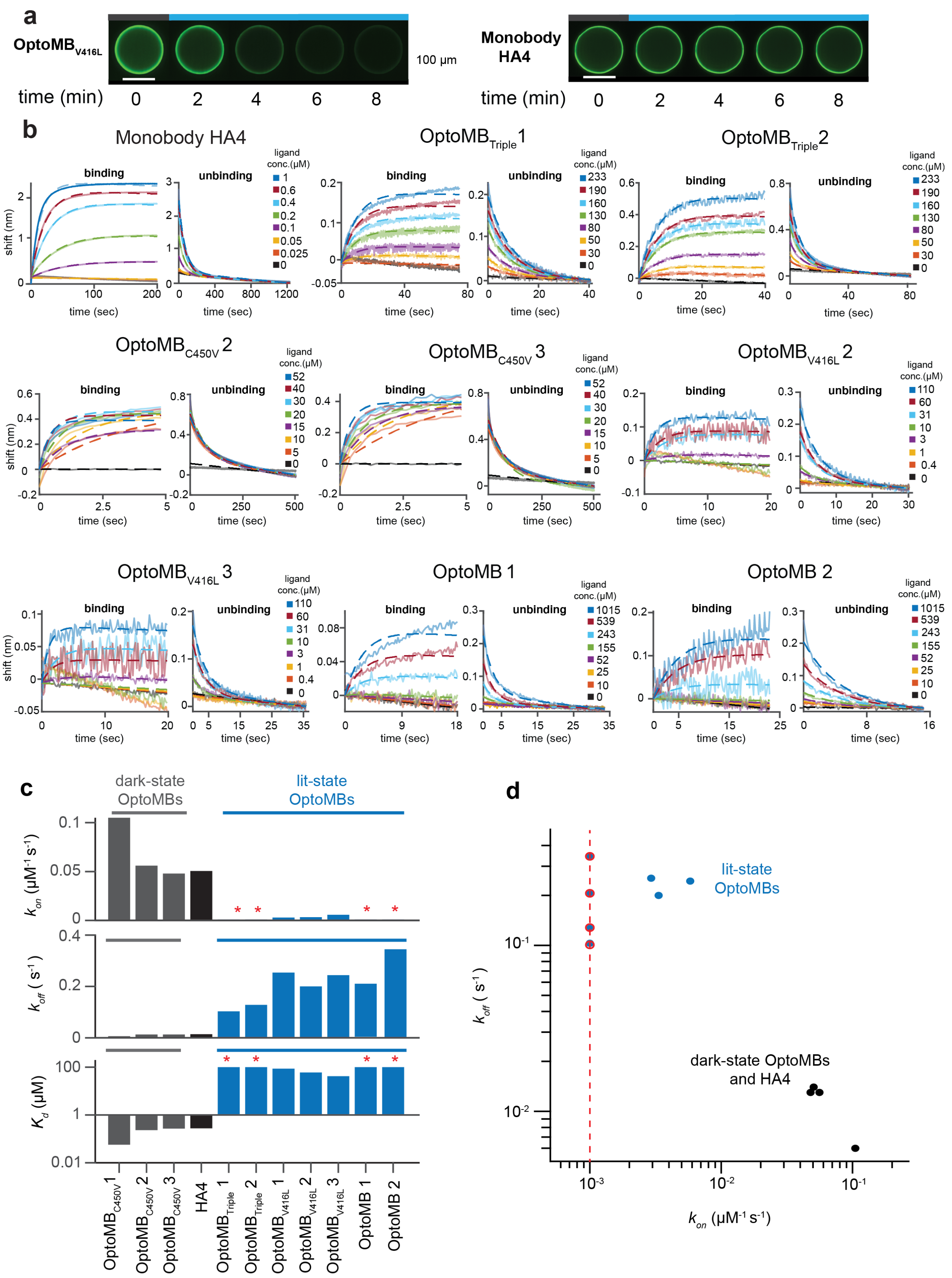
a
OptoMB V416L
100 µm
time (min) 0 2 4 6 8
Monobody HA4
time (min) 0 2 4 6 8
b
Monobody HA4
OptoMB Triple 1
OptoMB Triple 2
OptoMB C450V 2
OptoMB C450V 3
OptoMB V416L 2
OptoMB V416L 3
OptoMB 1
OptoMB 2
binding
unbinding
ligand conc.(µM)
shift (nm)
time (sec)
c
dark-state OptoMBs
lit-state OptoMBs
kon (µM-1 s-1)
koff (s-1)
Kd (µM)
OptoMB C450V 1
OptoMB C450V 2
OptoMB C450V 3
HA4
OptoMB Triple 1
OptoMB Triple 2
OptoMB V416L 1
OptoMB V416L 2
OptoMB V416L 3
OptoMB 1
OptoMB 2
d
lit-state OptoMBs
dark-state OptoMBs and HA4
koff (s-1)
kon (µM-1 s-1)

Supplementary Fig. 2: *In vitro* characterization of OptoMB binding. **a**, Time course of fluorescence microscopy images of YFP-SH2 binding to agarose beads conjugated with $\text{OptoMB}_{\text{V416L}}$ (left panel) or HA4 as control (right panel). Starting with beads incubated in the dark, time course begins upon blue light stimulation (t = 0), followed by a sequence of images taken every 2 min for a total of 8 min. Scale bar (white) represents 100 μm. **b**, BLI measurements of binding (left) and unbinding (right) of YFP-SH2 to immobilized monobody HA4, and several replicates for OptoMB, $\text{OptoMB}_{\text{C450V}}$, $\text{OptoMB}_{\text{V416L}}$ and $\text{OptoMB}_{\text{V416I\_G528A\_N538E}}$ in different light conditions (see Supplementary Table 2). **c**, Comparison of binding (k_{on}), unbinding (k_{off}), and dissociation (K_d) constants calculated from BLI measurements for different OptoMB variants, corresponding to dark (gray or black) or lit (blue) states, including replicates. The red asterisks highlight measurements of k_{on} below the limit of detection of 0.001 $\mu M^{-1}s^{-1}$. To calculate K_d values from measurements below this limit, we used k_{on} = 0.001 $\mu M^{-1}s^{-1}$. $\text{OptoMB}_{\text{Triple}}$ refers to the $\text{OptoMB}_{\text{V416I\_G528A\_N538E}}$ variant. **d**, Comparison of the binding and unbinding rate constants, k_{on} and k_{off}, for replicate measurements of the different OptoMB variants in their respective dark (black points) or lit (blue points) states. Measurements of k_{on} below the limit of detection (red dashed line) where assigned the limit k_{on} = 0.001 $\mu M^{-1}s^{-1}$ (red points). Source data are provided as a Source Data file.

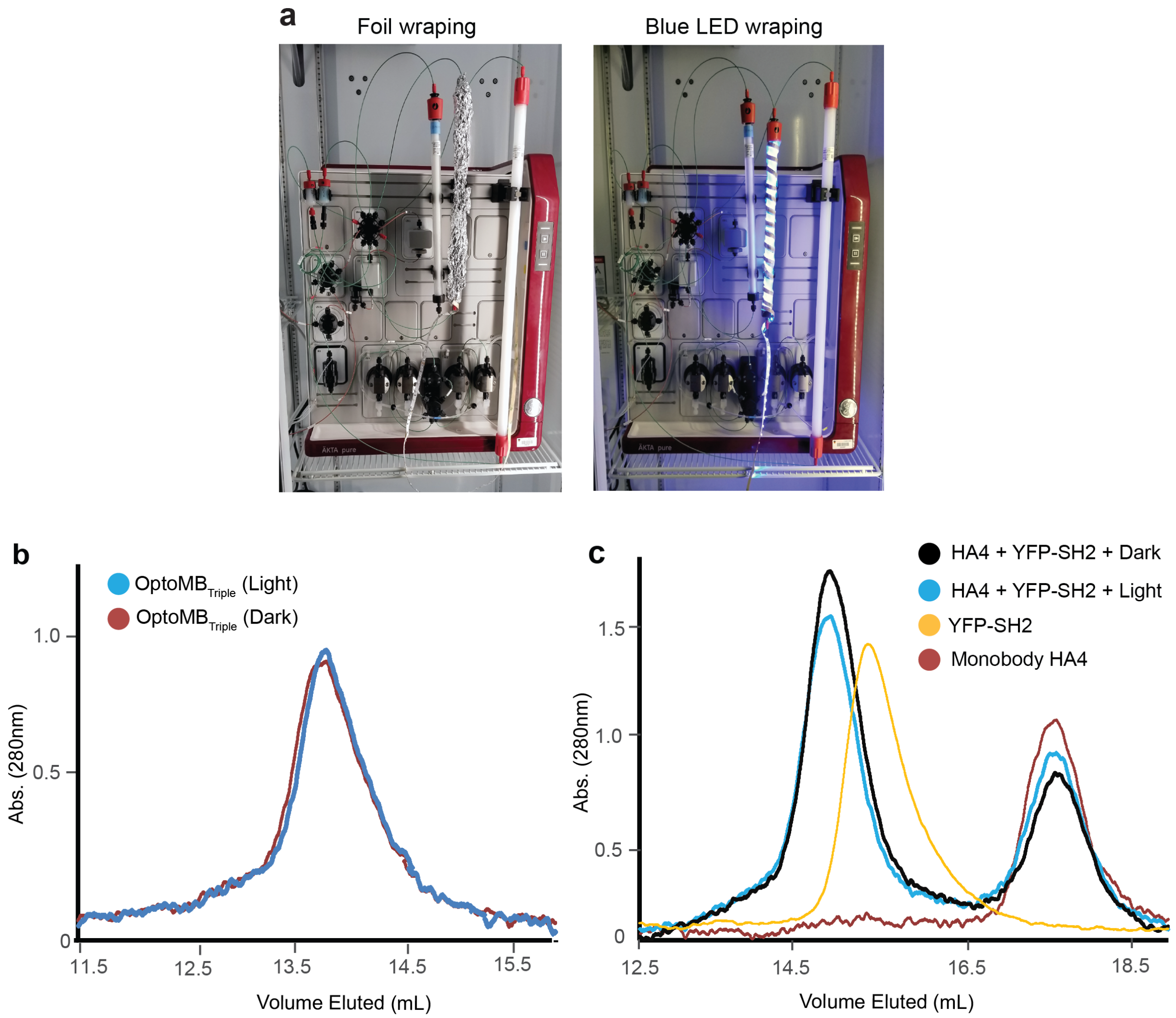

Supplementary Fig. 3: *In vitro* characterization of OptoMB binding in solution by size exclusion chromatography. a, Experimental setup for dark or lit size exclusion chromatography experiments using a Superdex 200 16/300 column (GE®). For experiments in the dark (left), the column was wrapped with thick aluminum foil, and the chromatography refrigerator covered with a black blanket to avoid light contamination (not shown). For experiments in the light (right), the column was wrapped with blue LEDs. **b**, Size exclusion chromatography control experiments for SUMO-tagged OptoMB$_{V416I_G528A_N538E}$ (OptoMB$_{Triple}$) in light (blue) and dark (red). **c**, Size exclusion chromatography control experiments to show identical elution profiles for the HA4-YFP-SH2 complex in dark (black) or light (blue) conditions. Purified YFP-SH2 (yellow) and HA4 monobody (red) are also shown as controls. Source data are provided as a Source Data file.

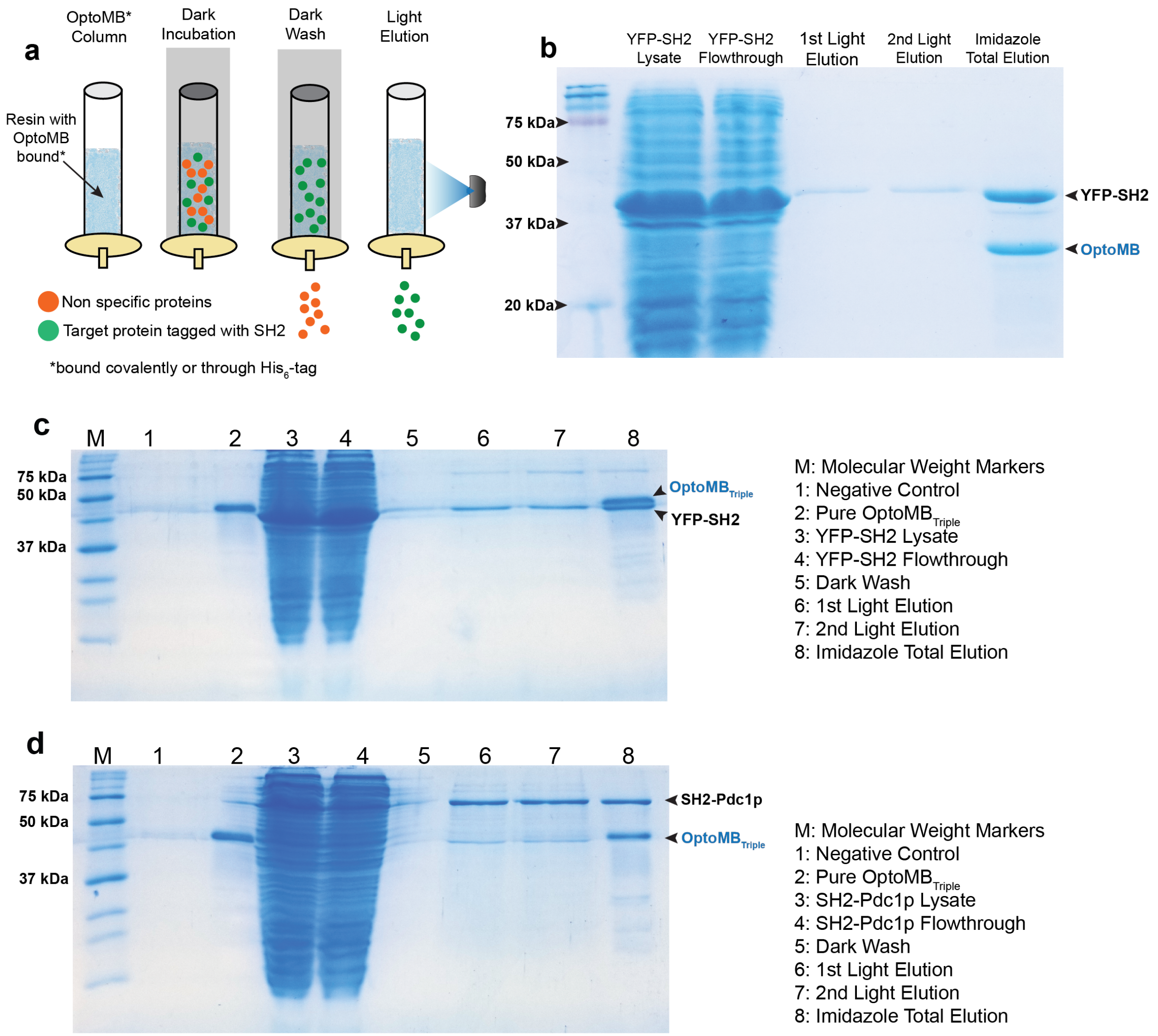

Supplementary Fig. 4: Light-Controlled Affinity Chromatography (LCAC) to purify SH2-tagged proteins using OptoMB immobilized on Co^{2+} agarose beads. a, Schematic diagram of LCAC procedure using a column packed with OptoMB-coated agarose beads. After flowing through crude extract and washing in the dark, elution is carried out by applying blue light to the surface of the column. **b**, SDS-PAGE gel of YFP-SH2 purified using a column packed with OptoMB agarose beads. **c**, SDS-PAGE gel of YFP-SH2 purified using a column packed with SUMO-tagged OptoMB$_{V416I_G528A_N538E}$ (OptoMB$_{Triple}$) agarose beads. **d**, SDS-PAGE gel of SH2-Pdc1p purified using a column packed with SUMO-tagged OptoMB$_{V416I_G528A_N538E}$ agarose beads. The "negative control" **c**,**d**, are final elutions from columns that were not exposed to crude extracts, but were otherwise treated the same. Purifications were repeated at least three times observing similar results.

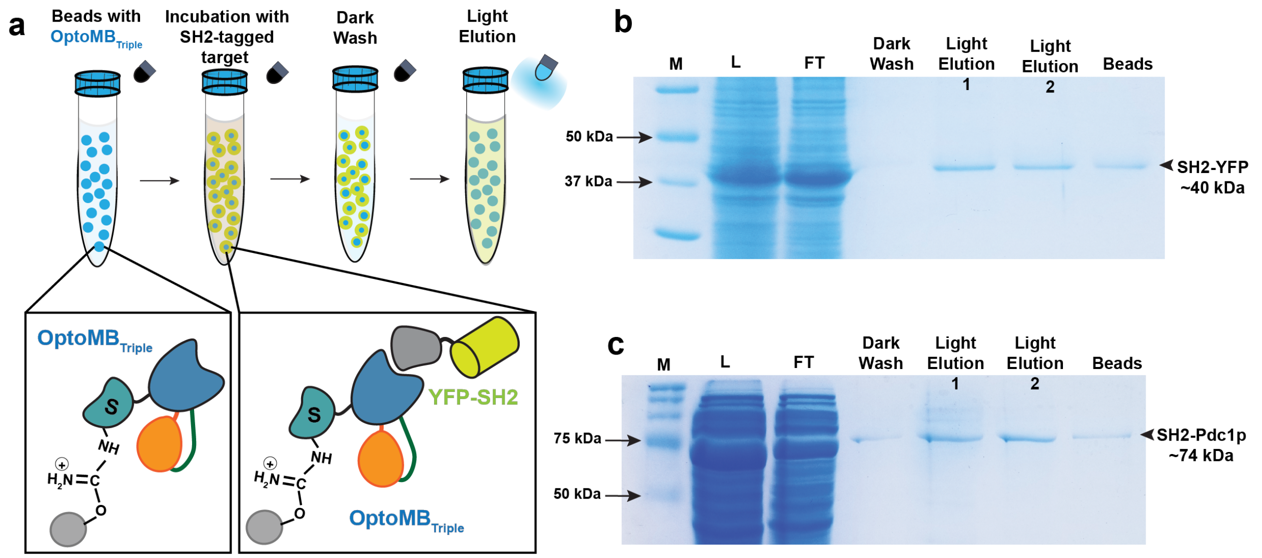

Supplementary Fig. 5: Light-controlled affinity chromatography to purify SH2-tagged proteins using cyanogen bromide (CNBr)-conjugated OptoMB. a, Schematic diagram of OptoMB$_{V416I_G528A_N538E}$ with SUMO tag (S) covalently linked to CNBr beads through surface-exposed primary amines, and their use in LCAC as described before. **b**, **c**, SDS-PAGE gel of YFP-SH2 (**b**) or SH2-PDC1 (**c**) purified using SUMO-tagged OptoMB$_{V416I_G528A_N538E}$ conjugated to CNBr beads, in batch. Molecular weight markers (M), lysate (L), unbound flow through (FT), washing step in the dark (Dark Wash), two consecutives light elution aliquots (Light Elution 1 and 2) and heat-treated beads resolved in SDS-PAGE gel (12% polyacrylamide). Purifications were repeated at least three times observing similar results.

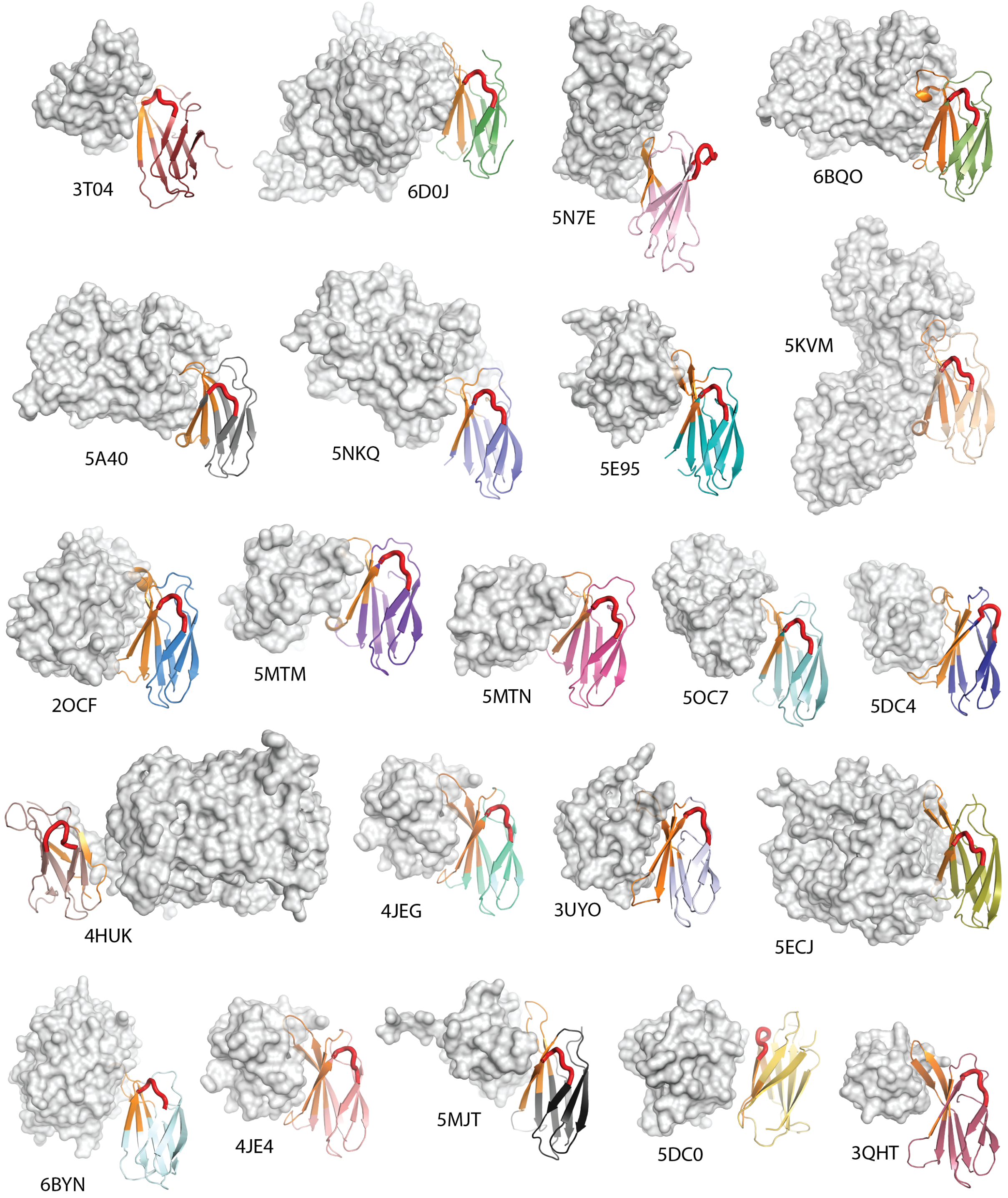

Supplementary Fig. 6: Structures of the other twenty-two unique monobodies displaying side-binding modes. Each monobody is shown (cartoons) in complex with their cognate targets (white surfaces) and labeled with their corresponding PDB code. The regions involved in target-binding interactions (orange) and the DE loop (red loop) where AsLOV2 is inserted in our OptoMB are highlighted.

Supplementary References

1. Koide, A., Abbatiello, S., Rothgery, L. & Koide, S. Probing protein conformational changes in living cells by using designer binding proteins: Application to the estrogen receptor. *Proc. Natl. Acad. Sci. U. S. A.* **99**, 1253–1258 (2002).
2. Wojcik, J. *et al.* A potent and highly specific FN3 monobody inhibitor of the Abl SH2 domain. *Nat. Struct. Mol. Biol.* **17**, 519–527 (2010).
3. Gilbreth, R. N. *et al.* Isoform-specific monobody inhibitors of small ubiquitin-related modifiers engineered using structure-guided library design. *Proc. Natl. Acad. Sci. U. S. A.* **108**, 7751–7756 (2011).
4. Koide, A., Wojcik, J., Gilbreth, R. N., Hoey, R. J. & Koide, S. Teaching an old scaffold new tricks: Monobodies constructed using alternative surfaces of the FN3 scaffold. *J. Mol. Biol.* **415**, 393–405 (2012).
5. Grebien, F. *et al.* Targeting the SH2-kinase interface in Bcr-Abl inhibits leukemogenesis. *Cell* **147**, 306–319 (2011).
6. Lu, M. *et al.* Structures of a Na+-coupled, substrate-bound MATE multidrug transporter. *Proc. Natl. Acad. Sci. U. S. A.* **110**, 2099–2104 (2013).
7. Sha, F. *et al.* Dissection of the BCR-ABL signaling network using highly specific monobody inhibitors to the SHP2 SH2 domains. *Proc. Natl. Acad. Sci. U. S. A.* **110**, 14924–14929 (2013).
8. Stockbridge, R. B., Robertson, J. L., Kolmakova-Partensky, L. & Miller, C. A family of fluoride-specific ion channels with dual-topology architecture. *Elife* **2**, 1–14 (2013).
9. Stockbridge, R. B. *et al.* Crystal structures of a double-barrelled fluoride ion channel. *Nature* **525**, 548–551 (2015).
10. Wojcik, J. *et al.* Allosteric inhibition of Bcr-Abl kinase by high affinity monobody inhibitors directed to the Src homology 2 (SH2)-kinase interface. *J. Biol. Chem.* **291**, 8836–8847 (2016).
11. Spencer-Smith, R. *et al.* Inhibition of RAS function through targeting an allosteric regulatory site. *Nat. Chem. Biol.* **13**, 62–68 (2017).
12. Nady, N. *et al.* ETO family protein Mtgr1 mediates Prdm14 functions in stem cell maintenance and primordial germ cell formation. *Elife* **4**, 1–28 (2015).

13. Zorba, A. *et al.* Allosteric modulation of a human protein kinase with monobodies. *Proc. Natl. Acad. Sci. U. S. A.* **116**, 13937–13942 (2019).

14. Last, N. B., Kolmakova-Partensky, L., Shane, T. & Miller, C. Mechanistic signs of double-barreled structure in a fluoride ion channel. *Elife* **5**, 1–10 (2016).

15. Salzman, G. S. *et al.* Structural Basis for Regulation of GPR56/ADGRG1 by Its Alternatively Spliced Extracellular Domains. *Neuron* **91**, 1292–1304 (2016).

16. Kükenshöner, T. *et al.* Selective Targeting of SH2 Domain–Phosphotyrosine Interactions of Src Family Tyrosine Kinases with Monobodies. *J. Mol. Biol.* **429**, 1364–1380 (2017).

17. Reckel, S. *et al.* Structural and functional dissection of the DH and PH domains of oncogenic Bcr-Abl tyrosine kinase. *Nat. Commun.* **8**, (2017).

18. Diver, M. M., Pedi, L., Koide, A., Koide, S. & Long, S. B. Atomic structure of the eukaryotic intramembrane RAS methyltransferase ICMT. *Nature* **553**, 526–529 (2018).

19. Gumpena, R. *et al.* Crystal structure of the human dual specificity phosphatase 1 catalytic domain. *Protein Sci.* **27**, 561–567 (2018).

20. McIlwain, B. C., Newstead, S. & Stockbridge, R. B. Cork-in-Bottle Occlusion of Fluoride Ion Channels by Crystallization Chaperones. *Structure* **26**, 635-639.e1 (2018).

21. Turman, D. L., Cheloff, A. Z., Corrado, A. D., Nathanson, J. T. & Miller, C. Molecular Interactions between a Fluoride Ion Channel and Synthetic Protein Blockers. *Biochemistry* **57**, 1212–1218 (2018).

22. Gupta, A. *et al.* Facile target validation in an animal model with intracellularly expressed monobodies. *Nat. Chem. Biol.* **14**, 895–900 (2018).

23. Last, N. B. *et al.* A CLC-type F-/H+ antiporter in ion-swapped conformations. *Nat. Struct. Mol. Biol.* **25**, 601–606 (2018).
