# Supplementary material for: Development of light-responsive protein binding in the monobody non-immunoglobulin scaffold
Source: Nat Commun. 2020 Aug 13;11:4045. doi: 10.1038/s41467-020-17837-7 (PMC7427095; doi:10.1038/s41467-020-17837-7)
Supplement: Supplementary file 2 — Description of Additional Supplementary Files [file 41467_2020_17837_MOESM2_ESM.pdf]

Description of Additional Supplementary Files

Supplementary Movie 1: Modeled Structure of the dark conformation of OptoMB. Rotation of the energy-minimized structural model of OptoMB shown in Figure 1e. The light-responsive chimera of AsLOV2 (orange), containing the Jα helix that undergoes structural rearrangement upon light stimulation (green), is inserted at position SS58 (in loop DE of HA4 monobody (blue), bound to SH2 domain (gray). Loop DE, in the monobody, connects βS2 (blue) with βS1 (black) forming the core of the monobody fold. Molecules are rotated 720 degrees in both y and x axis. Source data are provided as a Source Data file.

Supplementary Movie 2: Time-lapse imaging of Ni-NTA agarose beads coated with His-tagged OptoMB or the light-insensitive control OptoMB$_{\mathbf{C450V}}$. OptoMB coated bead (left) or control Monobody HA4 coated bead (right) were incubated with 2 μM of purified YFP-SH2 in solution (see Methods). Beads were imaged every 2 min using a 20X air objective and a blue LED light (450 nm) was turn on (indicated with the blue box) after beads were equilibrated in the dark. Time is indicated in hh:mm and the scale bar represents 50 μm.

Supplementary Movie 3: Time-lapse imaging of two Ni-NTA agarose beads coated with His-tagged OptoMB with spatial illumination of one bead. OptoMB coated beads were incubated with 2 μM YFP-SH2 in solution (see Methods). Imaging was done every 2 min using a 20X air objective. A masked square region of blue light (120 x 120 μm) was set to illuminate only the top right bead and the blue LED light (450 nm) was toggled on and off (indicated with the blue bar in the bottom left corner). Time is indicated in hh:mm and the scale bar represents 50 μm.

Supplementary Movie 4: Time-lapse imaging of HEK293T cells expressing OptoMB-irFP (shown) and membrane-localized SH2-mCherry-CAAX (not shown). Cells were imaged every 30 sec for 60 minutes using a 60X oil objective. Blue LED illumination (450 nm) was toggled on and off (as indicated by the blue box in the lower left corner). Time is indicated in mm:ss and the scale bar represents 10 μm.
