# Supplementary material for: Development of light-responsive protein binding in the monobody non-immunoglobulin scaffold
Source: Nat Commun. 2020 Aug 13;11:4045. doi: 10.1038/s41467-020-17837-7 (PMC7427095; doi:10.1038/s41467-020-17837-7)
Supplement: Supplementary file 7 — Reporting Summary [file 41467_2020_17837_MOESM7_ESM.pdf]

nature research

Corresponding author(s): Jared E.Toettcher and José L. Avalos

Last updated by author(s): Jul 6, 2020

# Reporting Summary

Nature Research wishes to improve the reproducibility of the work that we publish. This form provides structure for consistency and transparency in reporting. For further information on Nature Research policies, see our Editorial Policies and the Editorial Policy Checklist.

## Statistics

For all statistical analyses, confirm that the following items are present in the figure legend, table legend, main text, or Methods section.

| n/a | Confirmed | |
|---|---|---|
| ☐ | ☒ | The exact sample size ($n$) for each experimental group/condition, given as a discrete number and unit of measurement |
| ☐ | ☒ | A statement on whether measurements were taken from distinct samples or whether the same sample was measured repeatedly |
| ☒ | ☐ | The statistical test(s) used AND whether they are one- or two-sided<br>*Only common tests should be described solely by name; describe more complex techniques in the Methods section.* |
| ☒ | ☐ | A description of all covariates tested |
| ☐ | ☒ | A description of any assumptions or corrections, such as tests of normality and adjustment for multiple comparisons |
| ☐ | ☒ | A full description of the statistical parameters including central tendency (e.g. means) or other basic estimates (e.g. regression coefficient) AND variation (e.g. standard deviation) or associated estimates of uncertainty (e.g. confidence intervals) |
| ☒ | ☐ | For null hypothesis testing, the test statistic (e.g. $F$, $t$, $r$) with confidence intervals, effect sizes, degrees of freedom and $P$ value noted<br>*Give P values as exact values whenever suitable.* |
| ☒ | ☐ | For Bayesian analysis, information on the choice of priors and Markov chain Monte Carlo settings |
| ☒ | ☐ | For hierarchical and complex designs, identification of the appropriate level for tests and full reporting of outcomes |
| ☒ | ☐ | Estimates of effect sizes (e.g. Cohen's $d$, Pearson's $r$), indicating how they were calculated |

*Our web collection on statistics for biologists contains articles on many of the points above.*

## Software and code

Policy information about availability of computer code

| | |
|---|---|
| Data collection | All imaging data was acquired using Nikon NIS-Elements Version 4.4 software and visualized using Fiji (ImageJ). All Bio-layer interferometry data was acquired using Octet RED 96e instrument with 21 CFR Part 11 software. All size exclusion data was acquired on ÄKTA pure from GE® Healthcare running UNICORN 6.4 software. The gel band analysis was done using FIJI implementation (ImageJ) ( Version 2.0.0-rc-69/1.52o). |
| Data analysis | Change in fluorescence in cell based assays and their quantification was done using ImageJ v1.52p and analyzed using custom code written in MATLAB R2019a. Data for binding constants obtained using Bio-layer interferometry was analyzed using custom code written in MATLAB R2019a, with the code described in the methods section and attached as a supplementary file. All gel data was quantified in ImageJ. All code is available upon reasonable request. For OptoMB model Coot (model manual modification; Version 0.9) and YASARA (energy minimization; through Yasara Web Server) were used. |

For manuscripts utilizing custom algorithms or software that are central to the research but not yet described in published literature, software must be made available to editors and reviewers. We strongly encourage code deposition in a community repository (e.g. GitHub). See the Nature Research guidelines for submitting code & software for further information.

## Data

Policy information about availability of data

All manuscripts must include a data availability statement. This statement should provide the following information, where applicable:

- Accession codes, unique identifiers, or web links for publicly available datasets
- A list of figures that have associated raw data
- A description of any restrictions on data availability

The authors declare that all data supporting the findings of this study are available within the paper (and its supplementary information files). The source data underlying Table 2, Figures 1e, 2c,e, 3a,b,d, 5d,e, and Supplementary Figures 2b, and 3b,c are provided in the Source Data File.

# Field-specific reporting

Please select the one below that is the best fit for your research. If you are not sure, read the appropriate sections before making your selection.

☒ Life sciences ☐ Behavioural & social sciences ☐ Ecological, evolutionary & environmental sciences

For a reference copy of the document with all sections, see nature.com/documents/nr-reporting-summary-flat.pdf

# Life sciences study design

All studies must disclose on these points even when the disclosure is negative.

| | |
|---|---|
| Sample size | For live-cell imaging an objective lens magnification of ~60x was used to monitor protein translocation in as many cells as possible per field of view. As a result, typically sample size of n=5-10 cells was used per imaging run. |
| Data exclusions | No data were excluded. |
| Replication | All imaging data was acquired and reproduced multiple times. Protein purification based screen was performed once for each construct with further replication and characterization for the applicable hits. The reported Size exclusion measurements were acquired at least 3 times based on standards in the field after multiple trials to standardize protein concentrations . Measurements of binding constants were done in replicates for most of the constructs. LCAC was performed at least 3 times for each attached/conjugated |
| Randomization | Screening and testing was done for all samples the same way. Further characterization and optimization was done for samples the showed desired light response. |
| Blinding | All screening and testing of constructs was done with no bias. Otherwise, blinding was not relevant to this study. |

# Reporting for specific materials, systems and methods

We require information from authors about some types of materials, experimental systems and methods used in many studies. Here, indicate whether each material, system or method listed is relevant to your study. If you are not sure if a list item applies to your research, read the appropriate section before selecting a response.

## Materials & experimental systems

| n/a | Involved in the study | |
|---|---|---|
| ☒ | ☐ | Antibodies |
| ☐ | ☒ | Eukaryotic cell lines |
| ☒ | ☐ | Palaeontology and archaeology |
| ☒ | ☐ | Animals and other organisms |
| ☒ | ☐ | Human research participants |
| ☒ | ☐ | Clinical data |
| ☒ | ☐ | Dual use research of concern |

## Methods

| n/a | Involved in the study | |
|---|---|---|
| ☒ | ☐ | ChIP-seq |
| ☒ | ☐ | Flow cytometry |
| ☒ | ☐ | MRI-based neuroimaging |

## Eukaryotic cell lines

Policy information about cell lines

| | |
|---|---|
| Cell line source(s) | NIH 3T3 and HEK 293T cell lines were purchased from ATCC. |
| Authentication | None of the cell lines were authenticated beyond what was done by ATCC. |

| | |
|---|---|
| Mycoplasma contamination | Cell lines are periodically tested for mycoplasma (approx. once per year) and only negative-testing cell lines are used in the lab. |
| Commonly misidentified lines<br>(See ICLAC register) | No commonly misidentified cell lines were used in this study. |
